# Gut-larynx axis and its contribution to laryngeal immunity

**DOI:** 10.1128/msystems.01044-25

**Published:** 2025-10-07

**Authors:** Ran An, Elliott Xie, John Binns, Federico E. Rey, Christina Kendziorski, Susan L. Thibeault

**Affiliations:** 1 Department of Otolaryngology Head and Neck Surgery, School of Medicine and Public Health (SMPH), University of Wisconsin-Madison 5228, Madison, Wisconsin, USA; 2 Department of Biostatistics and Medical Informatics, SMPH, UW-Madison 5228, Madison, Wisconsin, USA; 3 Department of Bacteriology, College of Agriculture and Life Sciences, UW-Madison 5228, Madison, Wisconsin, USA; Northern Arizona University, Flagstaff, Arizona, USA

**Keywords:** gut microbiome, larynx, immunity, single cell, antibiotics

## Abstract

The larynx is vital for swallowing, breathing, coughing, and voice production, supported by its unique microbial and immunological environment. We hypothesized the existence of a gut-larynx axis, where resident gut and laryngeal microbiota influence immune modulation in the larynx. To test this, conventionally raised, wild-type C57BL/6 J mice were treated with an oral antibiotic regimen to disrupt gut microbiota and compared with untreated controls. Antibiotic treatment significantly disrupted gut microbiota but left laryngeal microbiota largely unaffected. However, antibiotic-treated mice showed notable changes in laryngeal epithelial and immune cells, as well as fibroblasts. Differential gene expression analysis revealed alterations in pathways related to epithelial barrier integrity, immune signaling, and bacterial response. Gene regulatory network analysis identified significant changes in regulons Etv4(+), Irf3(+), Hltf(+), Mga(+), and Nfil3(+). Additionally, cell-cell communication, particularly immune-epithelial interactions, was altered, with integrin-mediated signaling emerging as a key pathway. These findings suggest that gut and laryngeal microbiota may synergistically modulate immune responses, highlighting the importance of gut-larynx interactions in respiratory immunity.

**IMPORTANCE:**

This study investigates the gut-larynx axis, revealing how gut dysbiosis impacts immune responses in the larynx. Although laryngeal microbiota remained stable, significant immunological and cellular changes occurred following gut microbiota disruption. Transcriptomic alterations in epithelial integrity, immune signaling, and cell communication underscore the systemic impact of gut dysbiosis. The identification of integrin-mediated signaling as a key pathway in immune-epithelial interactions emphasizes the complexity of host-microbe dynamics. These findings suggest that gut health plays a critical role in shaping respiratory immunity, providing a foundation for future research into microbiota-driven immune modulation in the upper airway.

## INTRODUCTION

The role of gut microbiota in human health and disease has been well documented over the past two decades. Gut microbiota is particularly crucial for host immune development and modulation, influencing not only local immunity in the gastrointestinal tract but also systemic immune function (1–5). Numerous studies have demonstrated that gut dysbiosis—disruption of microbial balance—leads to various pathological intestinal conditions such as obesity (6, 7) and malnutrition (8), systemic diseases such as diabetes (9), chronic inflammatory diseases, such as inflammatory bowel disease (IBD) (10), and autoimmune diseases (11). Because of its multidirectional connection with extraintestinal organs, such as the lungs, brain, heart, kidneys, and skin, gut microbiota has been recognized as a “hidden organ” in our body (12).

The emerging concept of the gut-lung axis underscores a potential broader network of interactions between gut microbiota and the respiratory tract (13–16). Studies in both humans and animals have demonstrated that gut dysbiosis is linked to increased susceptibility to respiratory infections, impaired immune defenses, and exacerbated inflammatory responses in the lungs (13, 17). These effects can be mediated by gut-derived microbial metabolites, such as short-chain fatty acids (SCFAs), transported to the lungs, where these molecules promote differentiation of regulatory T cells, help maintain immune homeostasis, and suppress inflammatory responses (18, 19). Additionally, migration of immune cells primed in the gut, along with lipopolysaccharides (LPS) and other bacterial products, can enter systemic circulation, leading to alteration of immune cell populations or increased inflammation in the lung (14, 15, 20–23). However, the precise mechanisms underlying the interaction between the gut and lungs have yet to be fully understood.

The larynx, a portion of the upper respiratory tract, plays a vital role in regulating breathing, phonation, and airway protection during swallowing (24). It also serves as a critical anatomical and immunological gatekeeper between the upper and lower airways, ensuring that only filtered, conditioned air reaches the lungs (25, 26). The larynx functions as a critical barrier and sensory organ for inhaled environmental exposures and pathogens, enabling early detection and response to harmful stimuli (27, 28). Dysfunction of the larynx can therefore compromise pulmonary health by increasing the risk of aspiration, infection, or inflammation (29). It has a unique immunological architecture that protects against pathogens and external stimuli (30–32) and enables effective management of laryngeal disorders, such as benign vocal fold lesions, laryngitis, laryngeal papillomatosis, and even cancer (33–40). Prior studies from our lab and other research groups have demonstrated that this organ is a selective environment for microbial colonization, and distinct microbial communities significantly influence host immunity, contributing to the regulation of inflammatory responses and maintaining barrier integrity (30, 32, 41–44). Given the proximity of the larynx to the lungs and role as an immunological gatekeeper, we hypothesize the existence of a gut-larynx axis where gut microbiota work in concert with resident laryngeal microbes, contributing to the regulation of immune responses, in this critical region of the upper respiratory tract.

To test this hypothesis, we adopted a well-established oral antibiotic regimen in a mouse model (45, 46). In this model, normal mice are treated with a cocktail of antibiotics supplemented in their drinking water. We investigated the effects of gut microbiota disruption on microbial and immune landscapes of the larynx, at a single-cell resolution in the present study, aiming to expand the current understanding of local and systemic microbial-immune interactions and to offer insights into host-microbiota immune crosstalk and its implications for laryngeal health. The potential discovery of a gut-larynx axis could significantly reshape treatment paradigms for laryngeal disorders, facilitating non-invasive treatments such as probiotics or dietary adjustments.

## RESULTS

### Alteration of gut microbiota through antibiotic treatment in conventionally raised mice

Conventionally raised mice (*n* = 6), aged 8–10 weeks, were treated with an oral antibiotics regimen that included ampicillin, metronidazole, neomycin, and vancomycin for 2 weeks (45, 47). Throughout treatment, all antibiotic-treated (AB) mice remained alert with slightly fast breathing and reduced activity, while maintaining normal eyes, noses, and cheeks. Both male and female mice exhibited a slightly rough hair coat during treatment (Fig. S1A), likely due to the minor reduction in water intake. To mitigate dehydration, subcutaneous fluids were administered when needed. AB mice exhibited slight weight loss over the 2-week treatment, whereas the change was not significant compared with untreated control (CT) mice (Fig. S1B). A marked enlargement of the cecum was observed in AB mice after treatment, resembling cecal enlargement typically seen in germ-free (GF) mice (48–50) (Fig. S1C).

**Fig 1:**
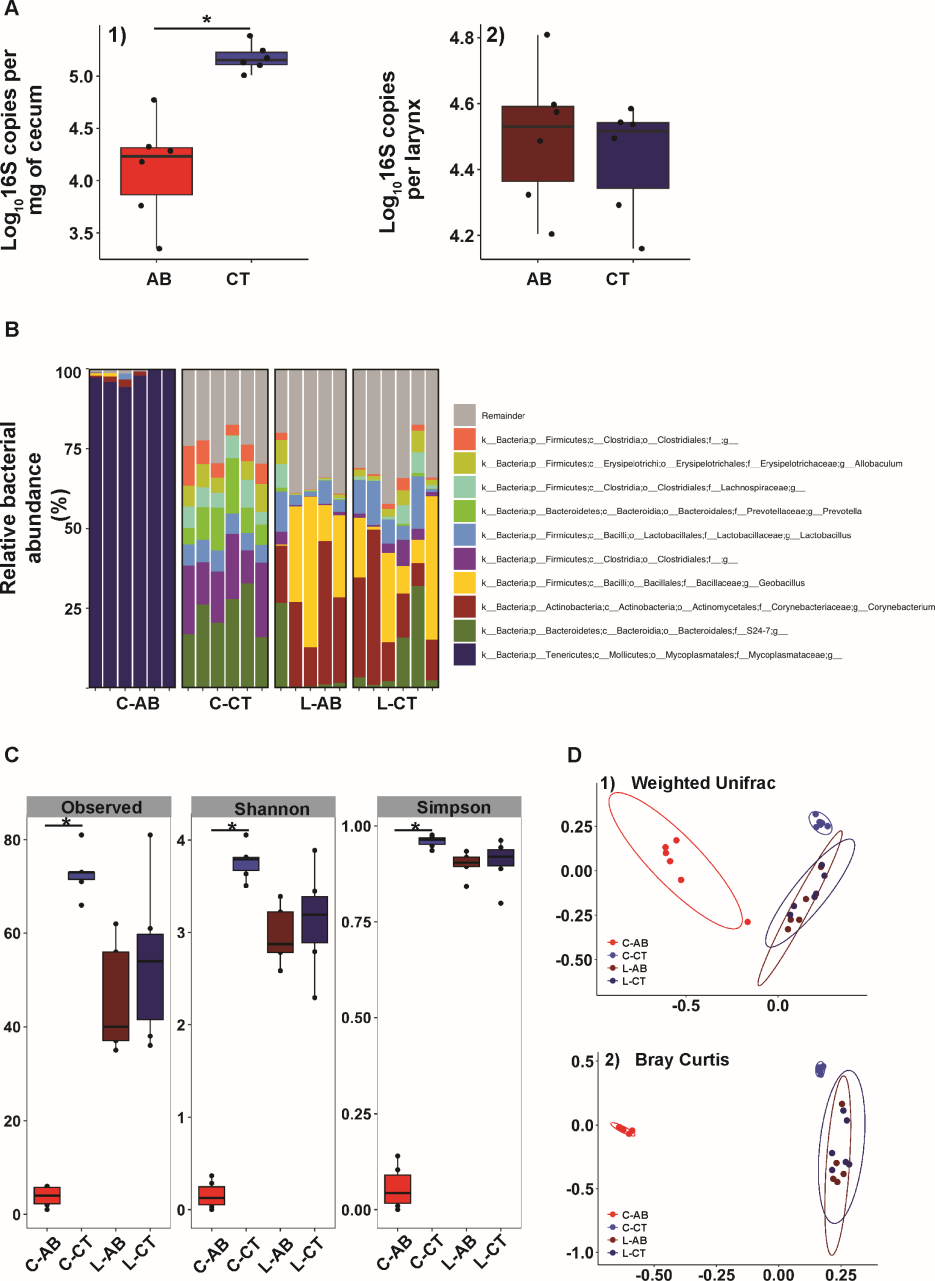
Characterization of gut and laryngeal microbiota in mice before and after AB treatment. (**A**) Total 16S rDNA copies per mg of cecum content (1) or per larynx (2) for antibiotic-treated (AB) and control (CT) mice. (**B**) Taxonomy compositions of microbiota at genus levels in AB and CT mice. C and L in C-AB/CT and L-AB/CT represent the cecum and the larynx, respectively. (**C**) Comparisons of gut and laryngeal microbiota α-diversity indices between AB and CT mice. (**D**) Principal coordinate (PCoA) analysis of β-diversity for gut and laryngeal microbiota in AB and CT mice using weighted UniFrac distance (1) and Bray-Curtis distance (2). PERMANOVA was applied to determine statistical differences in microbial community structure between groups (*P* = 0.001, F = 7.29). Dotted contours indicate groups obtained by comparisons with PERMANOVA.

Total bacterial abundance in both the larynx and cecum was assessed using 16S rDNA quantitative polymerase chain reaction (qPCR) in AB and CT mice (*n* = 6). A significant decrease in bacterial abundance was found in the cecum of AB mice, whereas no difference was observed in the larynx (Fig. 1A). Cecal and laryngeal microbiota were cultured on tryptic soy agar (TSA) plates supplemented with 5% sheep blood and incubated in aerobic conditions for 2–3 days. Colony-forming unit (CFU) of cecal aerobic/facultative bacteria was significantly lower in AB mice compared with CT mice, whereas no significant difference was observed in the CFU of laryngeal aerobic/facultative bacteria between the two groups (Fig. S1C). Prior work in our lab found that laryngeal samples yielded no colonies under anaerobic culture conditions, consistent with the larynx being predominantly colonized by aerobic/facultative bacteria. Anaerobic culturing, therefore, was not performed in the present study. However, we acknowledge that the limitation is that fastidious/non-culturable anaerobes cannot be excluded. Bacterial diversity and community structure were further analyzed using 16S rRNA gene sequencing. Significant alterations in the top 10 bacterial genera, as well as α and β diversity, were noted for the cecal microbiota of AB mice (Fig. 1B through D). In contrast, only a slight shift in the relative abundance of the top 10 genera of laryngeal bacteria was observed (Fig. 1B), and no significant changes in α or β diversity were detected in the laryngeal microbiota of AB mice. Our study successfully recapitulated the disruption of gut microbiota through AB treatment, with notable impacts on the cecal microbiota diversity and abundance (Fig. 1C and D). To further clarify the microbiota’s response to antibiotics and rule out potential confounding effects of gut microbiota, we performed separate PCoA analyses for gut and laryngeal samples (Fig. S1D). Consistent with previous observations, the laryngeal microbiota remained largely unaffected by the treatment. According to a bias-corrected ANCOM testing (ANCOM-BC), no differential abundance was detected at genus (Fig. S2) and other levels (data not shown) in the larynx between AB and CT mice; a group of bacteria predominated by an unknown genus of Mycoplasmataceae family was significantly enriched in AB mice cecum. This prompted us to further investigate the local immune profile in the larynx to explore potential changes in the immune profile as a consequence of the gut dysbiosis.

### Cell type heterogeneity in the larynx of AB-treated mice

Larynges from male and female mice, aged 8–10 weeks, were freshly collected, pooled by group (CT or AB), and dissociated into single-cell suspensions. We performed single-cell RNA sequencing (scRNA-seq) using a high-throughput, droplet-based platform. After removing low-quality cells, we obtained 40,373 cells from CT mice and 39,927 cells from AB mice for further analysis. Unsupervised graph-based clustering, followed by cell type annotation using *CellKb* and manual refinement based on the expression of canonical markers, identified 23 distinct cell types across four categories in both groups (Fig. S3A).

**Fig 2:**
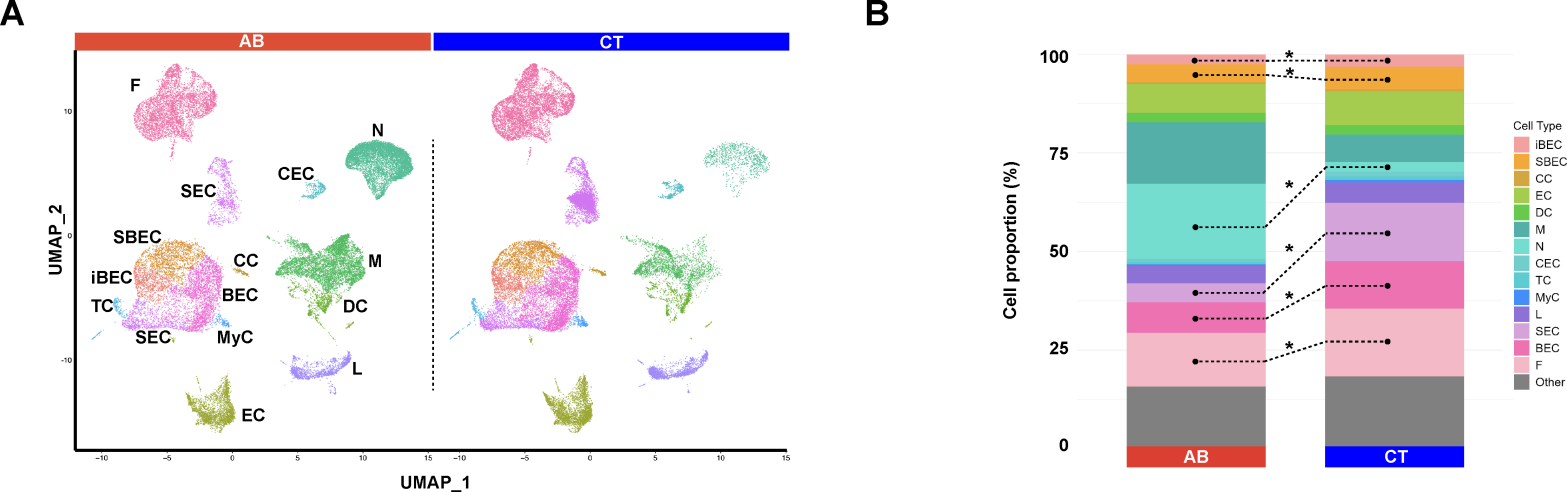
Proportions of cell types in antibiotic-treated (AB) and control (CT) mice. (**A**) Major cell types identified in AB and CT mice. (**B**) Proportion of each major cell type in AB and CT mice using scCODA. * indicates that the cell proportion is significantly different between AB and CT mice at FDR = 0.05.

Epithelial cells included basal epithelial cells (BEC), intermediate basal epithelial cells (iBEC), suprabasal epithelial cells (SBEC), secretory epithelial cells (SEC), ciliated epithelial cells (CEC), tuft cells (TC, also known as brush cells), cycling basal epithelial cells (cBEC), columnar cells (CC), and myoepithelial cells (MyC). Immune cells consisted of macrophages (M), dendritic cells (DC), neutrophils (N), and lymphocytes (L). Non-epithelial/immune cells included fibroblasts (F) and endothelial cells (EC), whereas miscellaneous cells encompassed chondrocytes (C), muscle cells (MC), Schwann cells (SC), skeletal muscle cells (SkMC), pericytes (P), smooth muscle cells (SMC), lymphatic endothelial cells (LEC), and red blood cells (RBC). Thymic epithelial cells (TEC), likely from residual thymus tissue surrounding the larynx, along with the miscellaneous cell types, are not discussed in this study due to their low representation. These findings are consistent with our earlier studies on the cell types identified in the mouse larynx (38). Despite the high similarity in cell type heterogeneity between treated and untreated mice, we observed notable differences in the proportions of several cell types between the two groups (Fig. 2A). Cell proportion of each major laryngeal cell type was compared between AB and CT mice using scCODA analysis (Fig. 2B). In AB mice, proportions of BEC, SEC, SBEC, and F were significantly reduced, whereas neutrophil proportions were markedly increased (FDR = 0.05). Unsupervised subclustering of major cell types revealed a varying number of subtypes in both groups. scCODA analysis of cell subtypes showed no significant differences in cell proportions between AB and CT mice at an FDR of 0.1. However, at a less stringent FDR of 0.3, we found significant decreases in fibroblast subtype 4 (F4), macrophage subtypes 4 (M4), and SBEC subtype 2 (SBEC2) in AB mice (Fig. S3B).

### Gut dysbiosis extensively altered gene expression in the larynx

**Fig 3:**
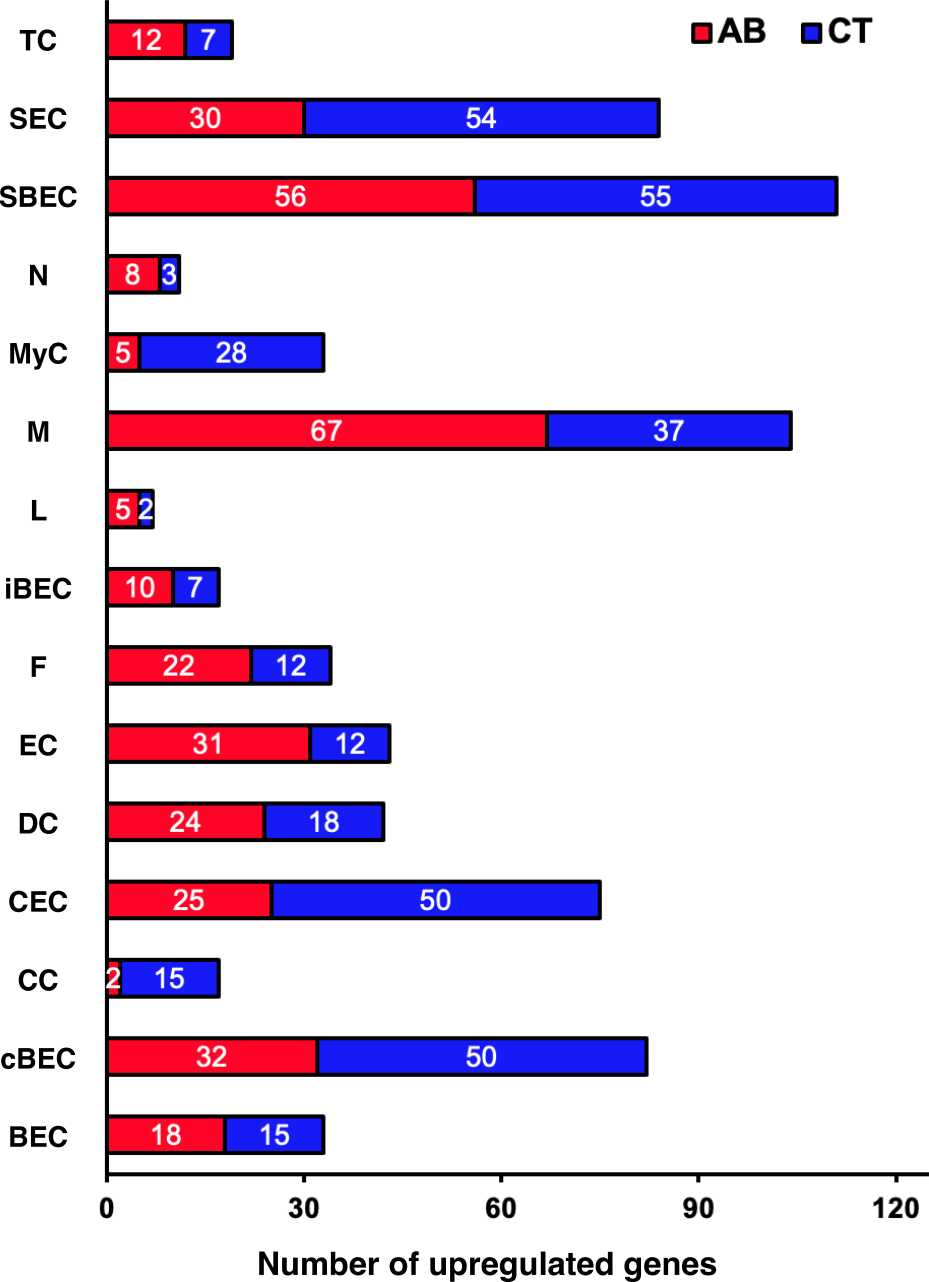
Number of differentially expressed genes (DEGs) upregulated in each major cell type in antibiotics-treated (AB) or control (CT) mice. BEC, iBEC, cBEC, CC, CEC, DC, EC, F, L, M, MyC, N, SBEC, SEC, and TC represent basal epithelial cell, intermediate epithelial cell, cycling basal epithelial cell, columnar cell, ciliated epithelial cell, dendritic cell, endothelial cell, fibroblast, lymphocyte, macrophage, myoepithelial cell, neutrophil, suprabasal epithelial cell, secretory epithelial cell, and tuft cell, respectively.

**Fig 4:**
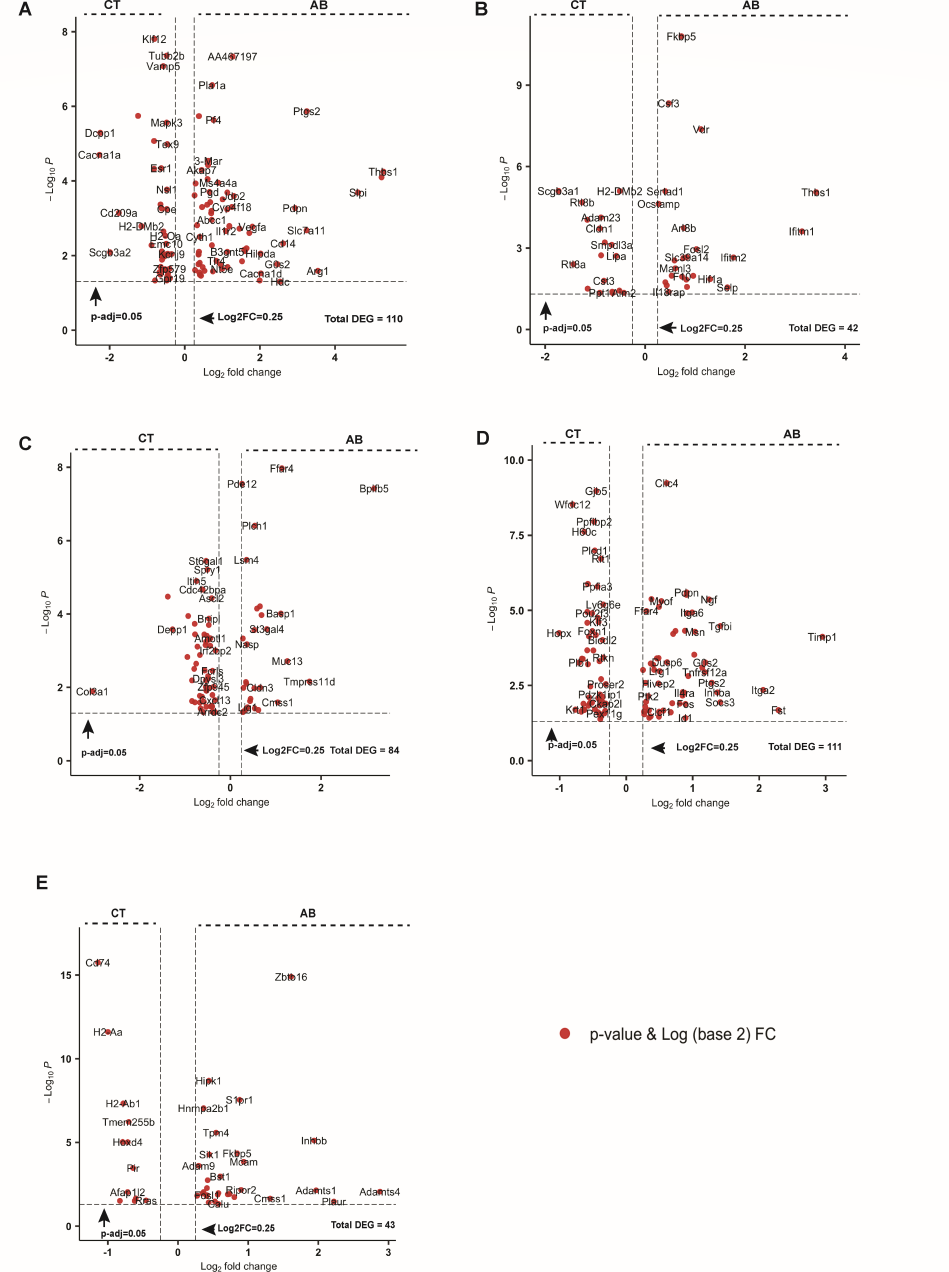
Volcano plots displaying the differentially expressed genes (DEGs) in macrophage (**A**), dendritic cell (**B**), secretory epithelial cell (**C**), suprabasal epithelial cell (**D**), and endothelial cell (**E**) of antibiotics-treated (AB) and control (CT) mice. Average log2 fold change > 0.250 or <−0.25, adjusted *P*-value < 0.05.

To investigate the impact of gut microbiota on laryngeal mucosa, at the transcriptomic level, we compared transcriptome profiles of larynges from AB and CT mice, analyzing both cell types and subtypes using a conservative pseudobulk method. Transcriptomes of all major cell types were affected to varying extents by gut dysbiosis (Fig. 3), with immune cells (M and DC), epithelial cells (SEC, SBEC, and cBEC), and EC showing the most pronounced changes. In macrophages, 110 genes were differentially expressed between AB and CT mice (avg_log2FC > 0.5, adjusted *P*-value < 0.05). Of these, 67 genes upregulated in AB mice were associated with biological processes such as cellular response to oxygen-containing compounds, negative regulation of intracellular signal transduction, regulation of angiogenesis, response to LPS, and regulation of protein serine/threonine kinase activity (Fig. 4A; Fig. S4A). In contrast, no significant Gene Ontology (GO) terms were identified for genes upregulated in the macrophages of CT mice under the same thresholds. Similarly, we identified 42 differentially expressed genes (DEGs) in DC (Fig. 3). In AB mice, upregulated genes were associated with processes including response to cytokine stimulus, regulation of protein kinase B signaling, regulation of DNA-templated transcription, and regulation of the viral life cycle (Fig. 4B; Fig. S4B). In CT mice, upregulated genes were linked to nervous system development, cell growth, and catabolic processes (Fig. 4C; Fig. S4B).

Transcriptomic profiles of laryngeal non-immune cells were impacted by gut dysbiosis to varying degrees, regardless of their distal anatomical location. In the SEC, 84 genes were differentially expressed between AB and CT mice (Fig. 3). Genes upregulated in AB mice were associated with epithelial structure maintenance, negative regulation of response to external stimuli, and autophagosome maturation. Conversely, genes upregulated in CT mice were linked to processes such as regulation of fat cell differentiation, synapse pruning, and regulation of epithelial to mesenchymal transition (Fig. 4C; Fig. S4C). In SBEC, 111 genes were differentially expressed (Fig. 3). Genes upregulated in AB mice were related to the regulation of apoptotic processes, cell population proliferation, multicellular organismal processes, leukocyte chemotaxis, and vasculature development (Fig. 4D; Fig. S4D). In contrast, genes upregulated in CT mice were involved in intermediate filament organization, Ras protein signal transduction, and synapse organization. EC also exhibited notable responses to AB treatment (Fig. 4E). In AB mice, genes involved in processes such as the transmembrane receptor protein serine/threonine kinase signaling pathway, DNA-templated transcription, and integrin-mediated signaling were significantly upregulated in EC (Fig. S4E). In CT mice, genes associated with positive regulation of angiogenesis, endothelial cell migration, interleukin-8 production, and endothelial cell-matrix adhesion were upregulated.

### Gut dysbiosis altered gene regulatory network in the larynx

The transcriptional state of a cell is governed by a gene regulatory network (GRN) where transcription factors (TFs) and cofactors collaborate to regulate the expression of target genes. To gain a deeper insight into the broader effects of AB treatment on the laryngeal transcriptome, we conducted single-cell regulatory network inference and clustering (SCENIC) analysis. This analysis was used to examine differences in the regulatory network of transcription factors (TFs) and their target genes, known as regulons, between AB and CT mice. This approach helps identify regulons that may influence the differentiation and function of key cell types in the larynx (43, 51). We assessed the activity of the top 10 regulons, quantified by area under the curve (AUC) scores, across 14 major cell types in both groups (Figs. S5 and S6). We identified a distinct set (approximately 33–91) of regulons unique to either AB or CT mice, along with a varying number (approximately 100–274) of regulons shared by both groups (Fig. S7). Active regulons and their actions were distinctly different between AB and CT mice, with regulon responses to AB treatment varying by cell type.

**Fig 5:**
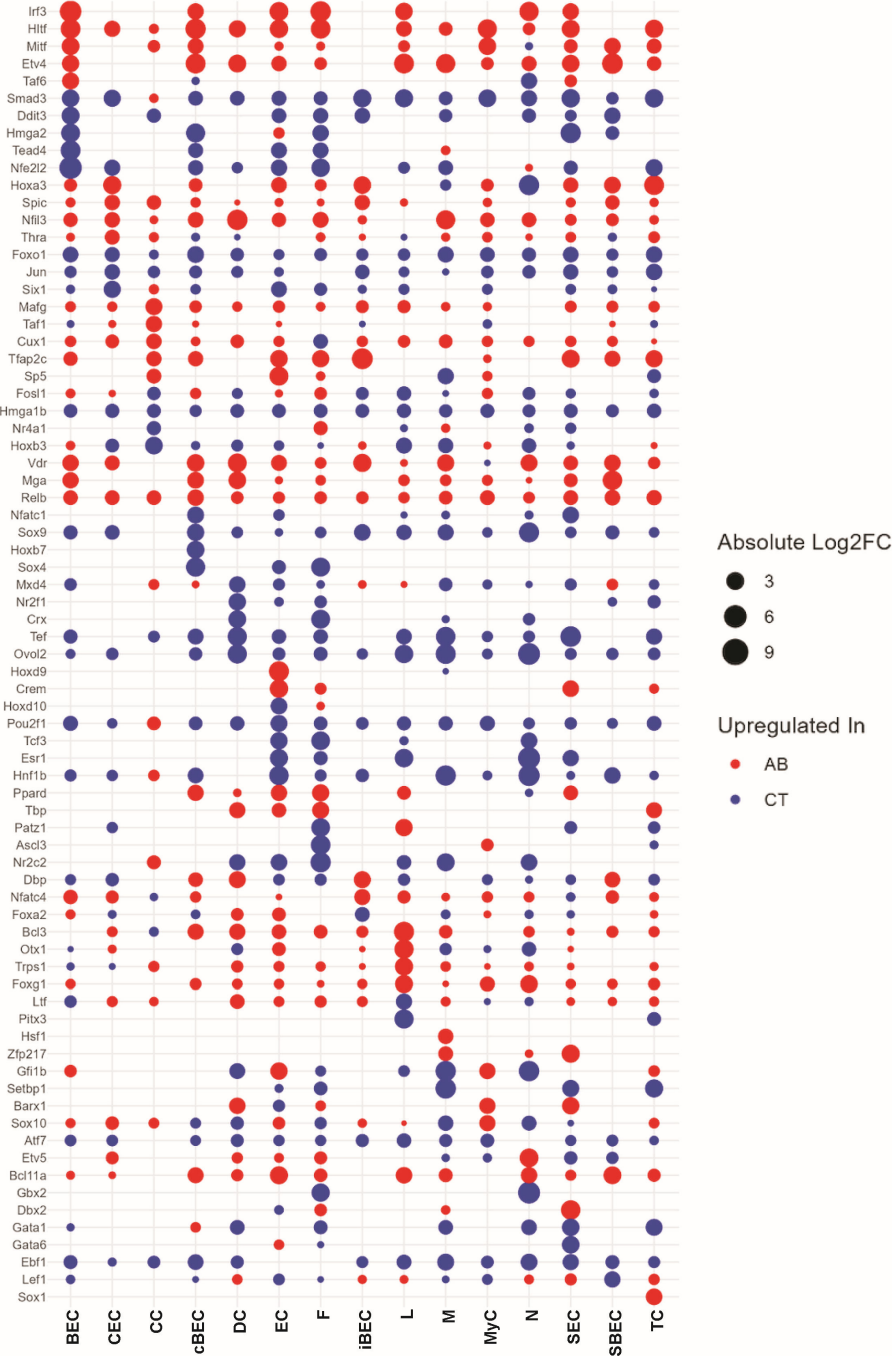
Top five differentially active regulons in antibiotic-treated (AB) and control (CT) mice (average log2 fold change > 0.25 or < −0.25, adjusted *P*-value < 0.05). BEC, iBEC, cBEC, CC, CEC, DC, EC, F, L, M, MyC, N, SBEC, SEC, and TC represent basal epithelial cell, intermediate epithelial cell, cycling basal epithelial cell, columnar cell, ciliated epithelial cell, dendritic cell, endothelial cell, fibroblast, lymphocyte, macrophage, myoepithelial cell, neutrophil, suprabasal epithelial cell, secretory epithelial cell, and tuft cell, respectively.

For each major cell type, we further identified differential regulons across mouse groups by evaluating the AUC scores demonstrating significant changes in response to AB treatment, as determined by the Wilcox rank-sum test (avg_log2FC > 0.25, adjusted *P*-value < 0.05). Given that immune cells (M, DC), epithelial cells (SEC, SBEC, and BEC), and EC had the strongest transcriptional responses to AB treatment, we further analyzed and compared the top five regulons in these cell types under both conditions. The following regulons appeared frequently among the top 5 regulons of multiple cell types in AB mice: Etv4(+), Irf3(+), Hltf(+), Mga(+), and Nfil3(+). Specifically, activity of Etv4(+), a regulon associated with cell differentiation, was significantly elevated in multiple cell types in AB mice, including BEC, SBEC, SEC, M, and DC (Fig. 5). A closely related TF, Etv5, showed increased activity in neutrophils from AB mice. It is worth noting that the proportion of all these cell types (except DC) changed in response to AB treatment. Regulon activity of Irf3(+) increased notably in BEC, SEC, M, and EC. Activity of the Mga(+) regulon, which is also involved in cell differentiation and proliferation, was markedly elevated in BEC, cBEC, SBEC, and DC in AB mice. Hltf(+), a regulon regulating G2/M transition in the cell cycle and playing roles in cell proliferation and apoptosis (52), demonstrated enhanced activity in multiple epithelial cell types and fibroblasts. Nfil3(+) activity was increased in phagocytes, specifically M and DC, in AB mice. In EC, the activity of Hoxd9(+) and Crem(+) regulons was significantly elevated. Hoxd9 plays a role in regulating morphogenetic pathways critical for bone and cartilage formation (53), and Crem modulates gene expression in response to cyclic AMP (cAMP) signaling, influencing cell proliferation, differentiation, and immune responses (53–55). Hoxd9 is also particularly important in regulating circadian rhythms, further underscoring its role in diverse physiological functions (56).

We compared the regulon specificity score (RSS) across mouse groups for each cell type to determine cell type specificity of regulons (Fig. S8). The top 10 regulons in the major cell types of AB mice were notably different from those in CT due to gut dysbiosis. Even the same regulon(s) present in both conditions exhibited a different RSS score(s). This evidence indicates TF-target genes' co-expression network shifts in response to AB and that transcriptional responses are cell type-specific.

### Gut dysbiosis altered cell-cell communication in the larynx

**Fig 6:**
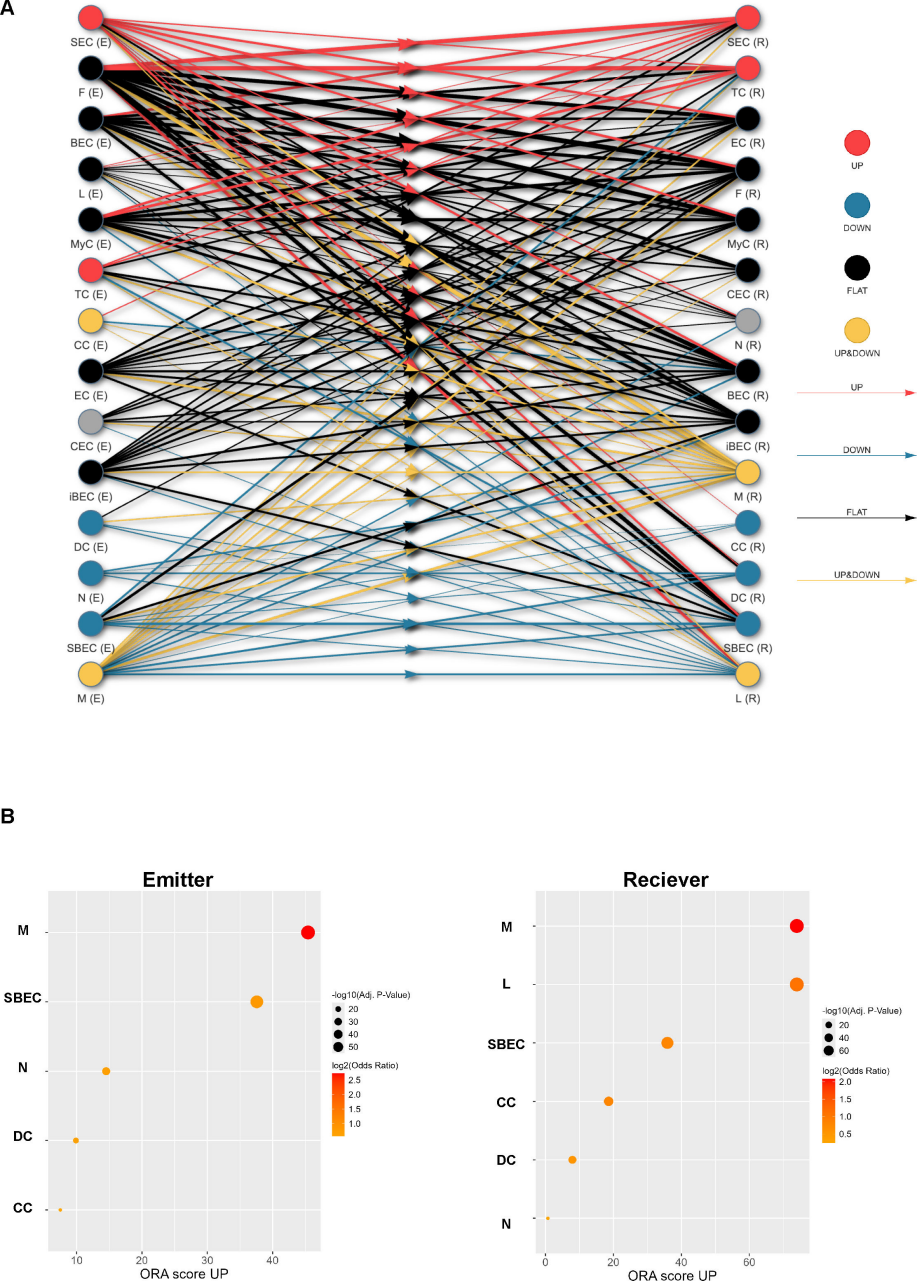
Over-representation of emitter cell types (E) and receiver cell types (R) in antibiotic-treated (AB) mice. (**A**) Cell-type networks with overrepresented emitter/receptor cell types and cell-type pairs that are upregulated (red), downregulated (blue), stable (black), or both up- and downregulated (yellow) in AB mice. When both upregulated and downregulated ligand-receptor interactions (LRIs) are significantly present between two cell types, the E/R cell type is yellow. Dots in the left (right) column represent emitter (receptor) cell types, and lines connecting two dots with arrows in the middle represent cell type pairs. (**B**) The top five emitter (left) or receptor (right) cell types in AB mice. Inf represents cell types with extremely small, adjusted *P*-value < 0.0001. Odds ratio (OR) quantifies how strongly a cell type is associated with an outcome. A higher log2(OR) indicates that the given cell type is more strongly associated with the condition. Overrepresentation analysis (ORA) was performed to identify whether emitter/receiver cell types appear more frequently in the data set than expected by chance. ORA score is defined by ORA score = −log2(OR) × log(adj.pval), where adj.pval represents adjusted *P*-value. A higher ORA score UP indicates a stronger statistical over-representation of the upregulated emitter/receiver cell type. CC, DC, L, M, N, and SBEC represent columnar cell, dendritic cells, lymphocyte, macrophage, neutrophil, and suprabasal epithelial cell.

The field of cell-cell interaction (CCI) inference is emerging and expanding rapidly. Cells communicate through various signaling molecules, such as hormones, cytokines, and neurotransmitters (57). CCI, the process by which cells exchange signals with each other to coordinate their activities, is essential for maintaining host biological functions. In this study, we inferred CCI in both mouse groups by identifying ligand-receptor interaction (LRI) pairs and comparing them across conditions to examine differential LRIs within each cell type (58). The cell type-centric over-representation analysis revealed that interactions between SEC, TC, CC, DC, N, SBEC, and M significantly changed across conditions (AB vs. CT) (Fig. 6A). Interactions involving ligands from SEC and TC and receptors on SEC, TC, EC, F, MyC, CEC, N, and SBEC were significantly upregulated in AB mice. In contrast, interactions with ligands from DC, N, and SBEC and receptors on SBEC, L, TC, M, CC, and DC were significantly downregulated in AB mice. Notably, both autocrine (self) and paracrine (other cell types) interactions of macrophages were either upregulated or downregulated in AB mice, suggesting that macrophages may be among the cell types most affected in intercellular communication. Accordingly, primary emitter and receptor cell types identified in our study were M, L, N, DC, SEC, and SBEC (Fig. 6B). We focused on these cell types to closely examine how their interactions were affected by gut dysbiosis.

**Fig 7:**
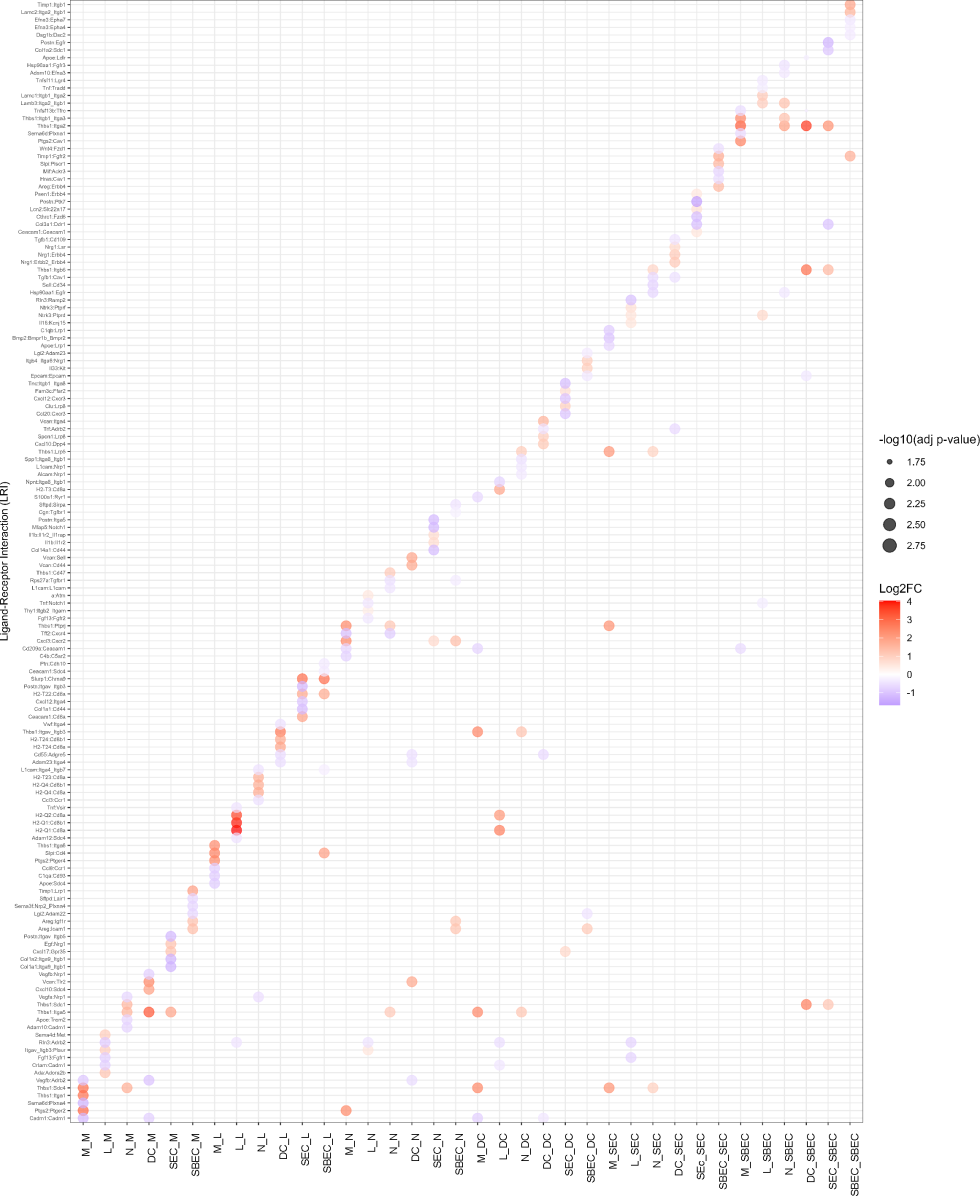
Top three ligand-receptor interactions (LRIs) of top emitter/receptor cell types in antibiotic-treated (AB) and control (CT) mice. Color bar represents log2 fold change level; dot size represents −log10(Adj. *P*-value, indicating significance of the LRIs. Red/blue dots represent LRIs that are more active in AB/CT mice. DC, L, M, N, SBEC, and SEC represent dendritic cells, lymphocytes, macrophages, neutrophils, suprabasal epithelial cells, and secretory epithelial cells.

The top three differentially active LRIs were identified for each of cell type pairs comprised of primary emitter/receptor cell types in both AB and CT mice (Fig. 7). Protein-protein network analysis, based on ligand and receptor genes identified in each of those primary emitter and receptor cell types, provided an overview of the interactions between ligands and receptors (Fig. S9). Functional enrichment analysis of these genes revealed distinct biological processes associated with LRIs in each cell type. In macrophages, LRIs were linked to cell proliferation, migration, response to stimuli, cell-cell communication, integrin-mediated adhesion, and apoptotic cell clearance (Fig. S10A). Lymphocyte-associated LRIs were primarily involved in antigen presentation, cell adhesion, response to external stimuli, and T cell activation (Fig. S10B). Neutrophil LRIs enriched processes like cell adhesion, inflammatory response, chemotaxis, and cytokine signaling (Fig. S10C). Dendritic cell LRIs were associated with cell migration, immune processes, and differentiation (Fig. S10D). In SECs, enriched processes included kinase regulation, apoptosis, and response to stress (Fig. S10E), whereas in SBECs, LRIs were linked to responses to endogenous stimuli and wounding, integrin-mediated adhesion, epithelial differentiation, and regulation of TGF-β production (Fig. S10F).

## DISCUSSION

This study investigated how AB-induced gut dysbiosis affects laryngeal immune function using single-cell transcriptomics. Wild-type, conventionally raised C57BL/6 J mice received a 2-week AB regimen in drinking, producing significant alterations in gut microbial diversity and community structure, whereas the laryngeal microbiota changed little over the observation window. Despite this, systemic effects were evident that gut dysbiosis exerted a broad, cell type-specific influence on the larynx, altering cell-type proportions and reshaping gene expression profiles, gene regulatory networks, and cell-cell communication in a cell-type-specific manner. Our findings, both here and in conjunction with our previous research (43), indicate that the gut and laryngeal microbiota together shape laryngeal immune function. 

Antibiotics, such as ampicillin, neomycin, metronidazole, and vancomycin (ANMV), are commonly used to disrupt gut microbiota in animal studies. Their effects on host physiology—cellular composition, signaling pathways, and organ function are well documented (47, 59, 60). We selected the ANVM regimen for its established treatment duration and ease of delivery. Josefsdottir et al. applied a similar regimen to probe microbiota-dependent hematopoiesis, showing multilineage alterations and suppression of multipotent progenitors after intestinal microbiota depletion (45). We reproduced gut microbiota disruption in C57BL/6 J mice, observing significant shifts in total and relative bacterial abundance, diversity, and community structure (Fig. 1; Fig. S1). Although direct antibiotic toxicity on host cells cannot be excluded (45, 46, 60), our data suggest minimal direct effects in this context: vancomycin and metronidazole are poorly absorbed and primarily act in the gut (Table S1), and we did not detect differential mitochondrial or ribosomal gene expression between AB and CT groups or enrichment of mitochondrial GO terms. Further studies are warranted to fully evaluate host responses.

AB-induced gut dysbiosis significantly altered cell-type proportions in AB mice, increasing immune cells—particularly neutrophils—and decreasing non-immune cells (BEC, SEC, SBEC, and F) proportion (Fig. 2A and B). The neutrophil increase contrasts with reports of reduced myeloid populations (macrophages, monocytes, and neutrophils) at systemic sites following AB treatment in germ-free mice (61). By contrast, basophil precursors can expand after AB treatment, elevating peripheral basophils and allergen responses (62). Several studies also report marked reductions in lymphocyte (B cells, CD4+ T cells, CD8+ T cells) in AB-treated mice (45), which we did not observe. These discrepancies may reflect the overgrowth of certain AB-resistant gut bacteria that promote innate immune recruitment, differentiation, and trafficking. In our study, Mycoplasmataceae dominated the gut after treatment. Members of this family (e.g., *Mycoplasma*, *Ureaplasma*) lack a cell wall and infect humans and animals, particularly in the respiratory tract (63–65). This is not uncommon, as AB regimens exert selective pressure favoring resistant taxa (66). AB-associated infections commonly involve *Staphylococcus aureus* and *Pseudomonas aeruginosa* in the intestine and extraintestinal organs (lungs, kidney, and liver) (60, 67). Although the larynx has low microbial biomass, commensals appear sufficient to influence immunity (43). Thus, a restructured gut community, even with reduced abundance comparable to the larynx, could affect circulating immune cell levels and, indirectly, laryngeal responses. Finally, inter-study variation in mouse strain, husbandry (diet, water, and housing), AB protocols (drug, dose, and duration), and method of cell composition analysis (scRNAseq, flow cytometry, and IHC/IF) likely also contributes to divergent outcomes and endpoint gut profiles. Meanwhile, circulating gut metabolites, like SCFAs, may influence the laryngeal epithelium. Although we did not measure SCFAs here, prior work shows SCFAs (butyrate, acetate, and propionate) can enhance barrier function and regulate epithelial cell differentiation and proliferation (68). We therefore speculate that altered SCFAs could relate to the reduced epithelial differentiation and proliferation we observed (BEC, SEC, and SBEC; Fig. 2A and B) and to Mycoplasmataceae overgrowth, warranting further targeted validation.

The gut microbiota has an extensive impact on the laryngeal transcriptome. In AB-treated mice, gut DEGs are largely downregulated versus controls (60). In our study, a notable number of DEGs were observed in each cell type between treated and untreated larynges (Fig. 3). As discussed, potential drivers likely include the depletion of gut microbiota, effects of surviving AB-resistant bacteria, and direct AB effects on host tissues. Although we did not profile gut gene expression, morphological and physiological changes we observed align with previous findings (Fig. 1; Fig. S1), suggesting that changes in the gut gene expression may influence the laryngeal transcriptome. AB treatment is known to induce oxidative stress in tissues (69), likely increasing the production of reactive oxygen species (ROS) in macrophages via mitochondrial stress or bacterial killing (Fig. 4A; Fig. S4A). However, no mitochondrial genes were identified among DEGs in macrophages or other cell types (Fig. 4A and 5A and C). Gut dysbiosis likely altered the immune milieu and macrophage signaling (Fig. S4A); for example, “response to LPS” was enriched in AB macrophages. Although Mycoplasmataceae lack a cell wall, immune activation could arise from lipoproteins/lipoglycans, affecting genes such as *Slpi*, *Nos2*, *Cd80*, *Adam9*, *Cd14*, *Cxcl3*, *Tlr4*, and *Pf4*. Protein kinase signaling was also enriched in macrophages and dendritic cells. Given the central role of kinases in immune regulation (70), this may reflect broader pathway shifts rather than macrophage-specific effects. Although intriguing, these findings remain correlative and should be interpreted cautiously.

Differential regulons likely shape laryngeal gene regulation at the transcriptomic level. Co-expression of TFs*—Etv, Irf3, Hltf, Mga*, and *Nfil3*—with their target genes across major cell types suggests that gut dysbiosis drives cell type-specific transcriptional programs (Fig. 5). *Etv4* (with *Etv5*) promotes keratinocyte differentiation, proliferation, and migration (71, 72). *Irf3* is a key antiviral TF regulating the type I interferon (IFN) responses during SARS-CoV-2 infection (73). In macrophages, *Irf3* activates distinct gene expression and induces apoptosis (74), often coordinating with *NF-*κ*B* and *AP-1* (75). *Hltf* participates in cell cycle checkpoints, DNA repair, and apoptosis. Mga is a dual-specificity TF influencing the MAX-network and T-box family. The MAX network, involving MYC and MXD/MAD proteins, governs cell proliferation, differentiation, apoptosis, and metabolism (76). *Nfil3* controls immune lineage development, such as innate lymphoid cells (ILC1,2,3) and T cells (Treg, Th17) (77, 78), and interfaces with the circadian clock to affect metabolism and cellular survival. Collectively, these regulons converge on pathways of differentiation, proliferation, and apoptosis in immune and non-immune cells, consistent with the cell proportion shifts observed. Notably, *Etv4* activity aligns with epithelial proliferation, whereas *Irf3* and *Hltf* are more associated with fibroblast proliferation in our data set.

Gut dysbiosis measurably altered intercellular communication (Fig. 6). Across cell types, enriched processes included integrin-mediated cell adhesion, response to stimuli, chemotaxis, apoptosis, and proliferation (Fig. S9 and S10). Integrin receptors (*Itgα1,2,3,4,5,6,9*, *Itgβ1,2,3,5,6*, *Itgm*, *Itgv*) were among the top differentially active LRPs in AB mice (Fig. 7). Integrin-mediated adhesion underpins immune defense and epithelial integrity. In immune cells, it enables endothelial transmigration and phagocytosis (79); in epithelial cells, it preserves tissue structure, promotes wound repair, and coordinates inflammation (80). Consistent with these roles, integrin signaling was enriched in EC from AB mice (Fig. 4E; Fig. S4E). The broader enrichment of LRPs linked to apoptosis, proliferation, chemotaxis, and stimulus response aligns with the observed shifts in cell proportions, suggesting the larynx adjusts signaling networks to maintain homeostasis under microbiota disruption.

### Conclusion

We conclude that AB-induced gut dysbiosis exerts a substantial, systemic influence on the larynx, particularly through cell type-specific alterations in cell proportion, gene expression, regulatory networks, and cell-cell communication at the transcriptomic level. Despite the limited impact on laryngeal microbiota composition, downstream effects on laryngeal immune regulation and cellular interactions are noteworthy. These results underscore the significance of the gut-larynx axis and suggest that gut microbiota may play a pivotal role in maintaining immune homeostasis and cellular function in distal organs. Future research should explore the long-term consequences of gut dysbiosis on laryngeal health and investigate potential therapeutic approaches targeting gut microbiota to modulate immune responses in the larynx and other distal sites. This study highlights the interconnectedness of gut and respiratory health and provides a foundation for further exploration into microbiota-driven regulation of systemic immunity.

### Limitations of the study

This study provides insight into the impact of AB-induced gut dysbiosis on laryngeal immunity, but certain limitations should be acknowledged. First, although our findings in the current and previous studies suggest that both gut and laryngeal microbiota influence laryngeal immune function, it remains challenging to delineate the specific contributions of each. The effects observed may arise from a synergistic relationship between the gut and laryngeal microbiota, making it difficult to quantify the unique role of the gut bacteria in laryngeal immune modulation. Second, AB treatment may also impact microbiota in other tissues and organs. Although gut microbiota exerts the most pronounced effect on host systemic responses, the role of microbiota in nearby organs—such as the lungs, pharynx, oral cavity, and nasal cavity—in shaping laryngeal immunity should not be overlooked. Although microbial changes in these regions were not assessed in this study, potential dysbiosis in these interconnected areas could significantly influence laryngeal immunity, highlighting the need for further investigation. Third, the small sample size (six mice per group) may limit statistical power, particularly in detecting subtle changes in laryngeal microbial communities. Although this sample size aligns with those used in many microbiome studies and was consistent with our a priori power analysis, we recognize that larger cohorts may strengthen the detection of low-abundance taxa and improve generalizability. Fourth, the potential for direct antibiotic toxicity on host cells cannot be excluded, and additional studies will be needed to fully evaluate direct host responses to antibiotics.

## MATERIALS AND METHODS

### Animals

All experiments were performed on 8- to 9-week-old mice to minimize variation in microbiota composition. Wild-type C57BL/6 J mice (Stock no. 000664) of both sexes were used for all experiments. Conventionally raised C57BL/6 J mice were obtained from the Jackson Laboratory and housed under specific-pathogen-free conditions at the Biomedical Research Model Services (BRMS) Laboratory. All mice were fed with autoclaved mouse breeder diet Labdiet 5021 (Purina, St. Louis, MO) and sterilized reverse osmosis water. Mice were bred and housed in the University of Wisconsin-Madison Biomedical Research Model Services Laboratory. Animal procedures were approved by the University of Wisconsin-Madison Institutional Animal Care and Use Committee and conducted in accordance with the National Institutes of Health Guide for the Care and Use of Laboratory Animals.

### Antibiotic treatment

Mice were fed with water *ad libitum* containing antibiotics: 0.5 g/L vancomycin (MilliporeSigma, Burlington, MA), 1 g/L ampicillin (MilliporeSigma, Burlington, MA), 1 g/L neomycin (MilliporeSigma, Burlington, MA), and 1 g/L metronidazole (MilliporeSigma, Burlington, MA). Flavoring (20 g/L grape-flavored Kool-Aid Drink Mix (Kraft Foods Global, Inc., Mendota Heights, MN) was added to the drinking water for both antibiotic-treated and untreated control groups. Treatment continued for 14 days (45).

### Sample collection

Mouse larynges were excised under sterile conditions using three designated sets of sterile surgical tools to prevent cross-contamination. All dissections were performed in a biosafety cabinet. The mouse was placed in a supine position. Using the first set of tools (forceps and scissors), the skin over the neck was carefully removed, and the thyroid glands were pushed laterally to expose the underlying trachea. A second set of tools was then used to access the oral cavity: the mouth was opened, the tongue gently pulled forward with forceps, and an incision was made along the right side of the jaw extending to the trachea. The procedure was repeated on the left side to fully disconnect the tongue from the jaw. A final cut was made at the upper trachea to remove the entire laryngeal structure en bloc, including the tongue, larynx, and a short portion of the trachea. The excised tissue was transferred to a sterile petri dish, and the larynx was isolated from surrounding tissues using a third set of sterile tools. To maximize access to the mucosal surfaces, the larynx was cut into approximately six pieces and transferred into a sterile 2  mL microcentrifuge tube containing 500  µL sterile PBS. The tube was vortexed at maximum speed for 5–10 min to dislodge mucosal-associated bacteria. Tissue fragments were then carefully removed using sterile forceps or pipette tips. The bacterial suspension was centrifuged at 15,000 rpm for 5 min to pellet bacterial cells. The supernatant was discarded, and the resulting pellet was preserved at –80°C until further processing. Cecum contents were collected into sterile 2 mL microcentrifuge tubes and kept on ice for no more than 40 min prior to processing. Wet weights were determined by weighing the tubes before and after sample collection (81). Cecum content collected from individual mice was preserved at −80°C until processing.

### Evaluation of bacterial total abundance

SYBR green quantitative polymerase chain reaction for 16S rDNA gene was performed with 515F/806R primers on genomic DNA extracted by Qiagen kit from laryngeal tissue and cecum contents. A standard curve was established to determine the copy number of the 16S rDNA gene following the method in Nadkarni et al. (82).

### 16S rRNA gene sequencing

Based on a power analysis using G*Power (83), we estimated that a sample size of 6 mice per group provides 80% power at α = 0.05 to detect an effect size of 1.6 (Cohen’s *d*), consistent with prior observations in similar antibiotic treatment studies (83–86), for detecting microbiome differences in α diversity metrics. For β diversity, power analysis was performed with vegan, an R package, using a resampling-based approach, in which Bray-Curtis or Weighted UniFrac dissimilarity matrices were repeatedly subsampled at varying sample sizes, followed by permutational analysis of variance (PERMANOVA) testing, to estimate the probability of detecting a significant group difference (*P* < 0.05) under the effect size of 0.8. Larynges were collected from conventionally raised mice treated or untreated with AB, minced into 0.5 mm pieces on a sterile Petri dish under a dissection scope. Bacterial cells were harvested as described above in “Sample collection.” DNA extraction from the bacterial cells, 16S rRNA gene sequencing, and data analysis followed the method described in An et al. (43). Specifically, the bacterial cell pellet was digested in an enzymatic lysis buffer consisting of 20 mM Tris-Cl, 2 mM EDTA, 1.2% Triton X-100, and 20 mg/mL lysozyme for 1 h at 37°C. DNA extraction was subsequently performed using Qiagen DNeasy Blood & Tissue kit (Qiagen, Germantown, MD) following the manufacturer’s instructions. V4 regions of the 16S rRNA gene were amplified using DNA-free Platinum Taq DNA Polymerase (ThermoFisher Scientific, Waltham, MA) in a 25 µL reaction containing 15 ng of DNA template and 400 nM of 515F/806R primers fused with Illumina sequencing adapters. Non-template PCR control (NTC; *n* = 3) and a PCR-positive control with *Helicobacter pylori* genomic DNA as template were included for each PCR run. PCR cycling conditions were as follows: one cycle of enzyme activation at 95°C for 3 min, followed by 35 cycles of denaturation at 95°C for 30 s, annealing at 55°C for 30 s, and extension at 72°C for 30 s, and a final extension at 72°C for 5 min. Each sample was amplified in triplicate. Resulting PCR products were identified on 2% agarose gel, and the successful reactions were pooled, purified with NucleoSpin Gel and PCR cleanup kit (Takara, San Jose, CA), and quantified with Qubit dsDNA HS Assay Kit (Invitrogen, Eugene, OR). Samples were equimolar pooled and sequenced on the Illumina MiSeq 2 × 250 bp platform. NTC was confirmed to have no visible amplicon bands and was excluded from subsequent analysis. Given that the larynx is a low microbial biomass sample, an extraction negative control (ExNeg, *n* = 3) containing only sterile PBS was processed in parallel with the laryngeal tissues to control the potential environmental contamination. No bands were observed for the extraction negative controls; therefore, these controls were not included in sequencing.

Sequences were processed using the QIIME2 pipeline (87). Demultiplexed 250 bases paired-end sequences were imported using Casava 1.8 format and denoised using DADA2 to obtain an amplicon sequence variant (ASV) table (88, 89). Singletons (ASV present <2 times) and ASVs that are present in less than 10% of the samples were discarded. Any ASV with a total abundance across all samples below 1% of the total abundance of all ASVs across all samples was removed. Greengenes reference sequences (clustered at 99% similarity) were used to train a naïve Bayes taxonomy classifier to further annotate ASVs taxonomically (90). ASVs were then collapsed based on the genus level. ASV files were imported and visualized in RStudio (v4.1.2). Alpha-diversity (observed ASV richness, Simpson, and Shannon diversity) and beta-diversity (Bray-Curtis and Weighted UniFrac) analyses were performed using the q2-diversity plugin at a rarefaction depth of 3,000 sequences per sample. Microbial community differences between groups were evaluated through principal coordinates analysis (PCoA) and statistically examined by PERMANOVA test at *P* = 0.001.

### Single-cell RNA sequencing and analysis

We included six biological replicates per condition, with each replicate representing a pool of four mouse larynges. Dissociation of laryngeal tissue, droplet-based scRNA-seq of the single-cell suspensions, single-cell data analysis including quality control, pre-processing, dimensionality reduction, clustering and subclustering, and cell type annotation was performed following the paper by An et al. (43), with the exception of the mitochondrial ratio cutoff set to 0.1. Single-cell library was sequenced at NovaSeq SP1 flow cell using a 150 × 150 bp sequencing reaction targeting >60,000 reads/cell, yielding a calculated depth of over 150K mean reads per cell, which was sufficient for identifying cell populations and estimating gene expression statistics.

#### Differential expression and enrichment analysis across conditions

To elucidate the differentially expressed (DE) genes between conditions in a specific cell type, a pseudobulk method was performed. This approach considered the inherent correlation structure in the data. We aggregate the cellular expression profiles within each sample to mitigate the effects of individual cellular variability. Subsequently, DESeq2 was used as the primary tool to compare expression levels. This methodological choice allowed for a robust and statistically sound identification of DE genes. In this comparison, DE genes with avg_log_2_FC >0.25 or <0.25 and adjusted *P*-value <0.05 were deemed significant. Mitochondrial genes (genes that begin with “mt-”) and ribosomal genes (genes that begin with “Rps-”) were removed, prior to further analysis, from the DE genes, given that these genes are not of interest to us in the present study. A list of the DE genes (avg_log_2_FC > 0.25, adjusted *P*-values < 0.05, unless otherwise specified) was analyzed for enrichment of common biological process (BP) using the R package enrichR (version 3.0) (91). Enrichment results were visualized with bar plots using defined functions provided in the R script.

#### Gene regulatory network analysis

To construct gene regulatory networks (GRN), single-cell regulatory network inference (SCENIC, v0.2.0) was used to identify TFs and associated target genes, referred to as regulons, in each cell type of AB and CT groups (51). A regulon activity score (RAS) was calculated for each regulon in each single cell by summing up the area under the recovery curve. In short, SCENIC calculates enrichment of a regulon as an area under the recovery curve, where the recovery curve plots gene expression of all genes in a cell (*x*-axis) vs. the number of genes recovered from the regulon (*y*-axis). The regulon specificity score (RSS) for a cell type was then calculated according to the entropy of RAS of cells within the cell type compared to other cell types. An RSS ranges from 0 to 1, with a higher value representing greater specificity of a regulon in the cell type. To reduce computation time and effectively increase the data quality, for cell (sub)types with more than 20 cells, we randomly average every 20 cells, so that each group produced a pseudo-cell by the average gene expression profile of cells within the group, and SCENIC was applied to the pseudo-cells. Differential regulon activity between conditions was assessed using the Wilcoxon rank-sum test, as implemented in Seurat.

#### Comparison of cell proportions

To identify significant differences in cellular composition between AB and CT samples, we employed single-cell compositional data analysis (scCODA), a Bayesian model specifically designed for comparing cell type proportions in single-cell data while accounting for the compositional nature of the data and controlling for sample-specific covariates (92). False discovery rate (FDR) cutoff was set to 0.05 and 0.3. To examine compositional changes at the subcluster level, we first extracted cells belonging to each major cell type separately and then performed scCODA analysis on their respective subclusters using the same FDR threshold.

#### Cell-cell communication analysis

To investigate potential differences in intercellular communication patterns between AB and CT samples, we utilized scDiffCom, a statistical framework for comparative cell-cell communication analysis using single-cell transcriptomics data (58). Using default parameters, scDiffCom analyzed ligand-receptor interactions between all pairs of cell types and identified significant changes in communication patterns between the two conditions. The analysis incorporated known ligand-receptor interactions from curated databases and evaluated their differential activity based on the expression levels of interacting molecules. The interaction of ligand-receptor pairs and their enrichment analysis for cell types was performed using StringDB (string-db.org).

## Data Availability

ScRNA-seq data are accessible in Gene Expression Omnibus under the accession number GSE290718. 16S rRNA gene sequencing data are accessible in GenBank under BioProject PRJNA1230173. Other data and analytical methods are available from the corresponding author upon request. R markdown and Python notebook scripts enabling the main steps of the analysis performed are available from the corresponding author on request.

## SUPPLEMENTAL MATERIAL

Supplemental figures msystems.01044-25-s0001.pdf Figures S1 to S10.

Legends msystems.01044-25-s0002.docx Supplemental figure legends.

Supplemental table msystems.01044-25-s0003.docx Table S1.

ASM does not own the copyrights to Supplemental Material that may be linked to, or accessed through, an article. The authors have granted ASM a non-exclusive, world-wide license to publish the Supplemental Material files. Please contact the corresponding author directly for reuse.
