# Supplementary figures and images for: Gut-larynx axis and its contribution to laryngeal immunity
Source: mSystems. 2025 Oct 7;10(11):e01044-25. doi: 10.1128/msystems.01044-25 (PMC12625719; doi:10.1128/msystems.01044-25)

A

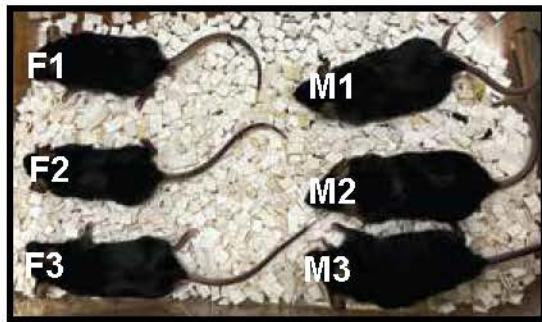

B

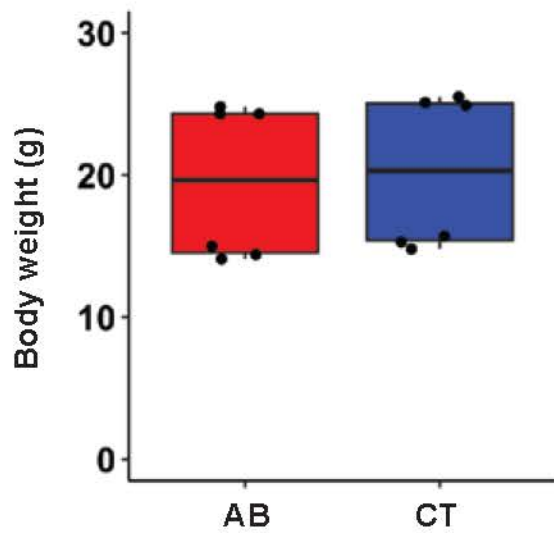

C

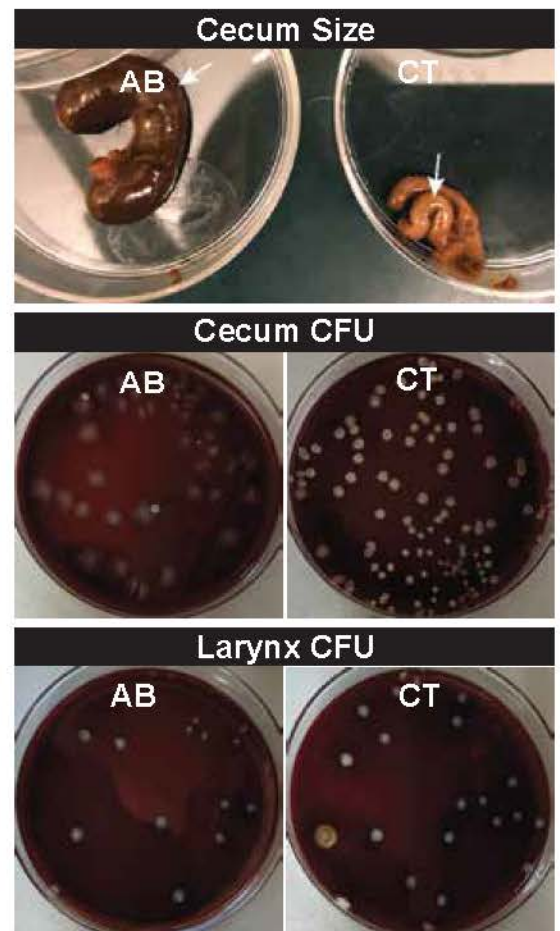

D

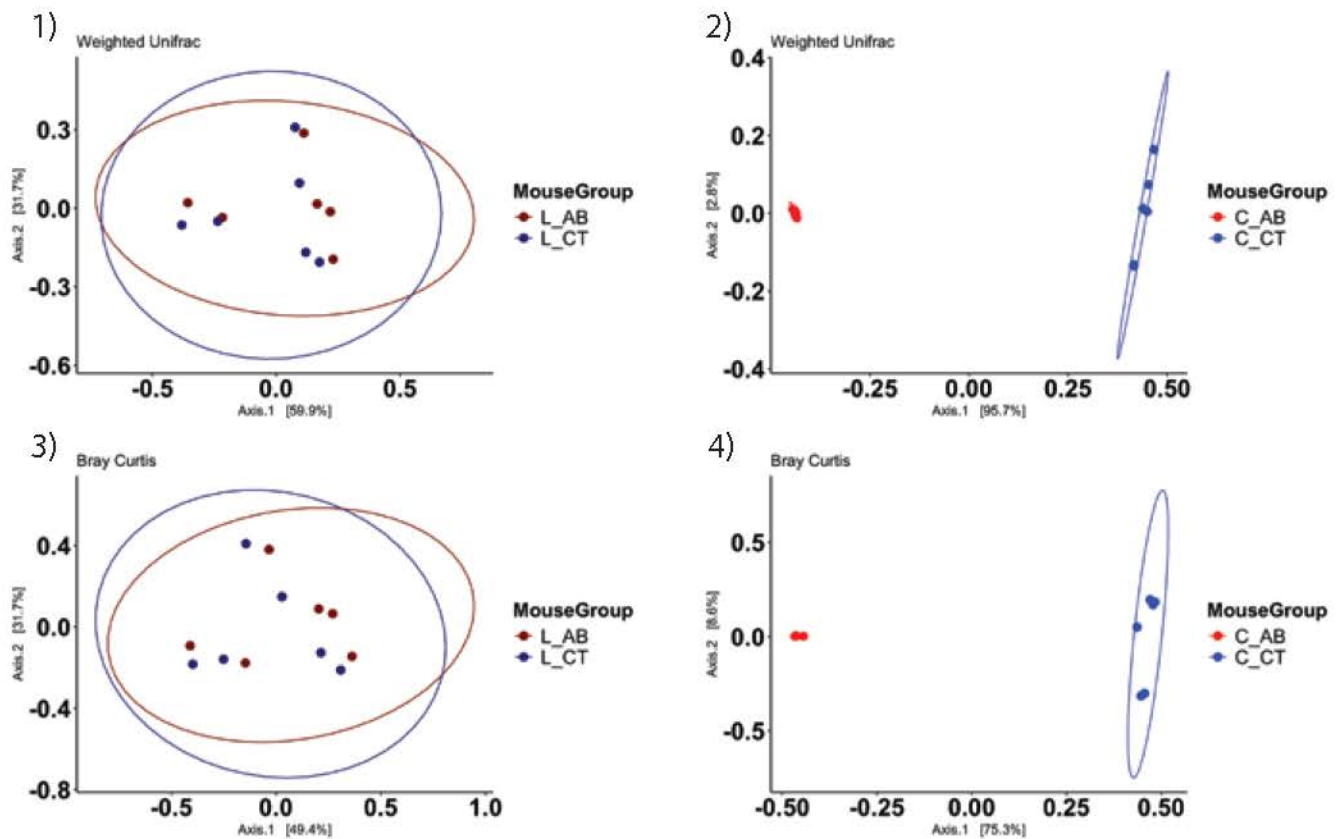

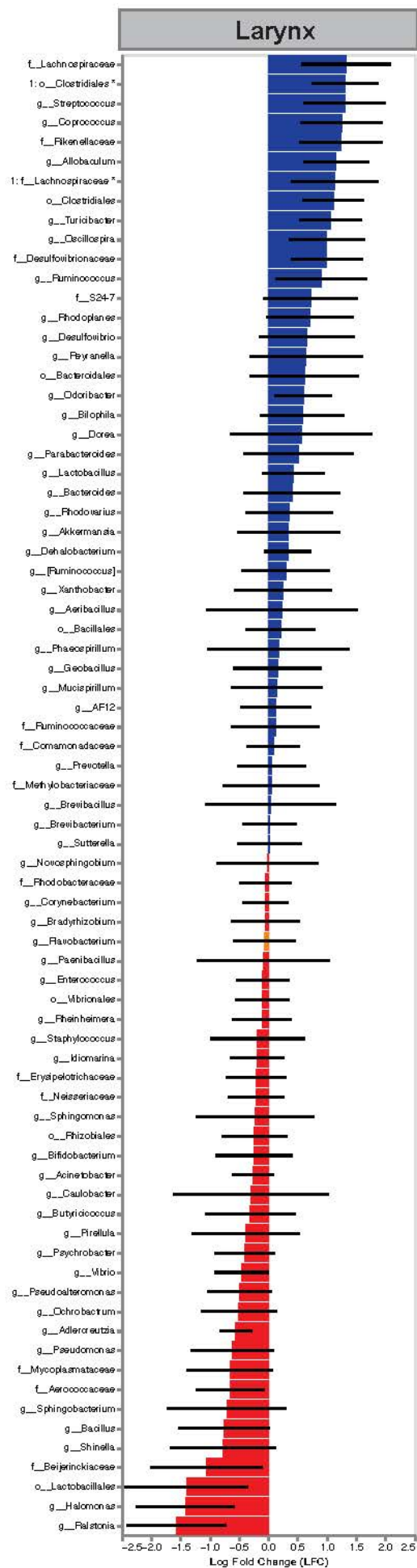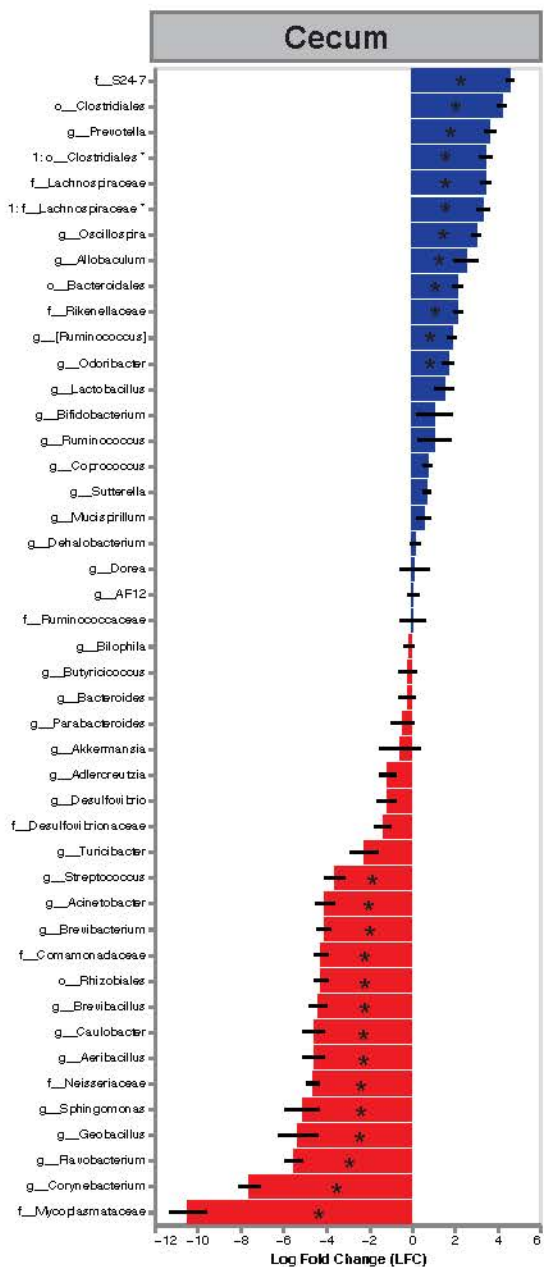

MouseGroup

CT

AB

**A**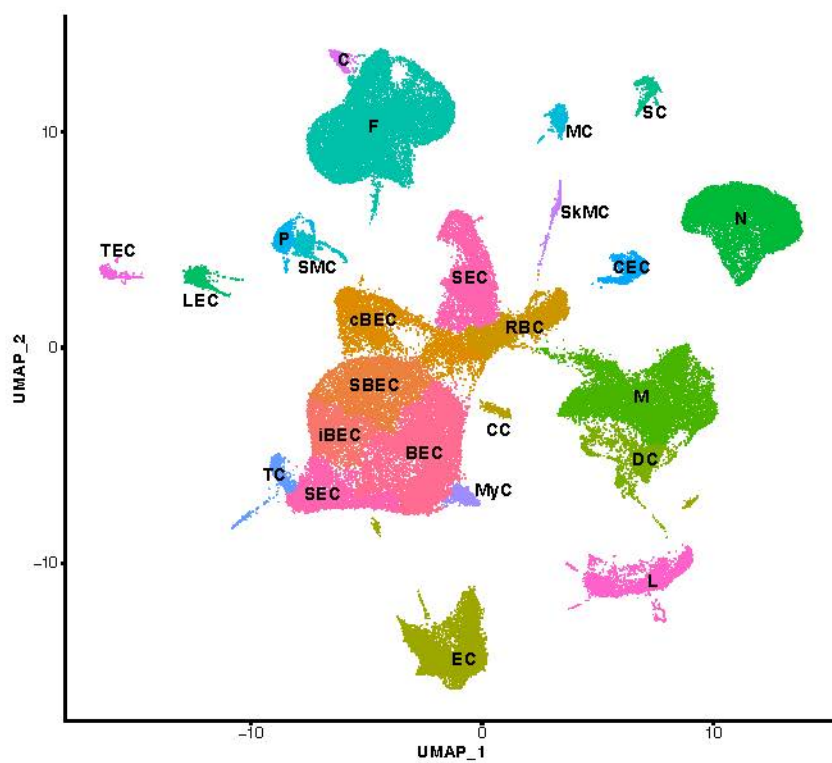**B**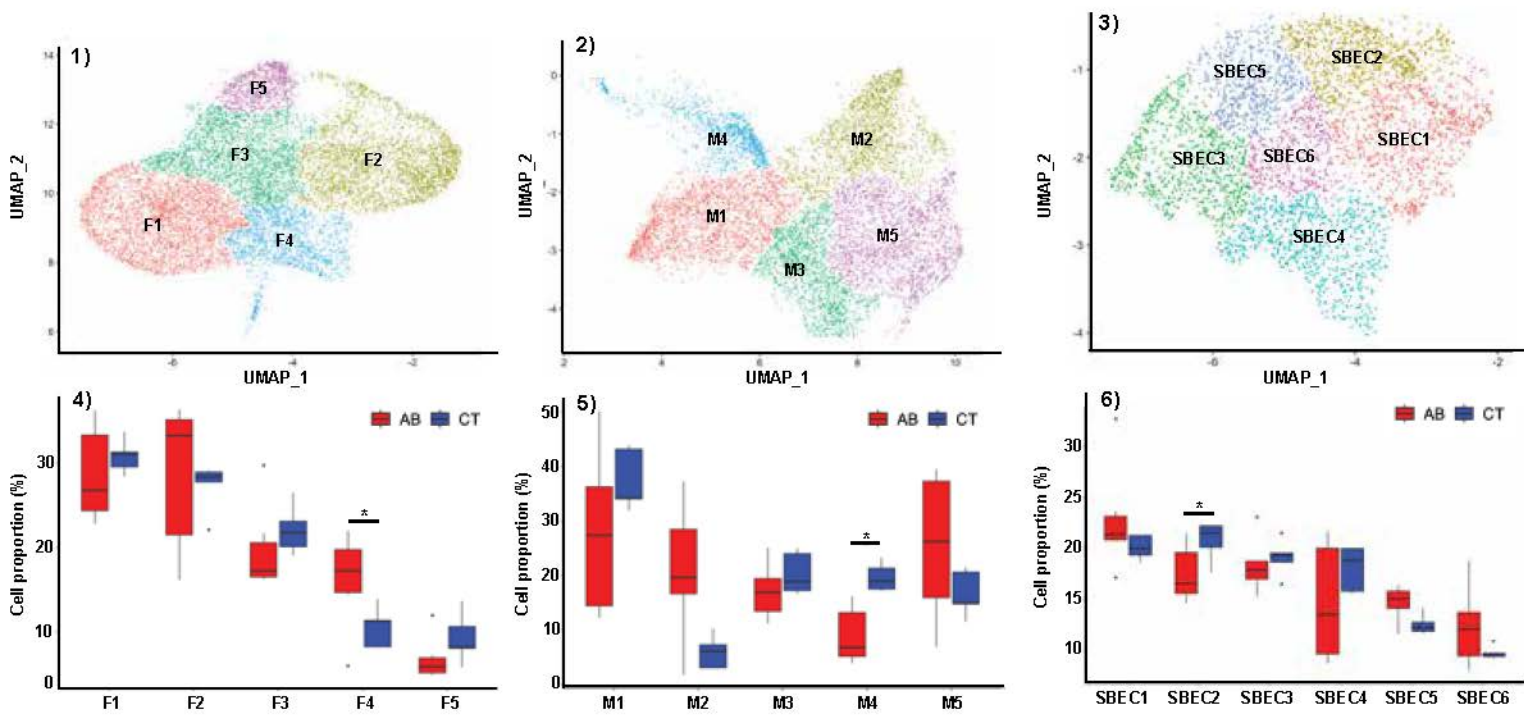

A

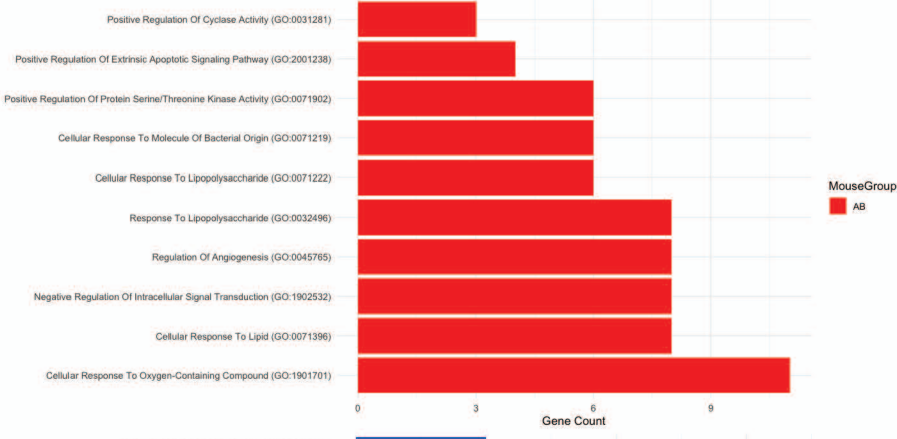

B

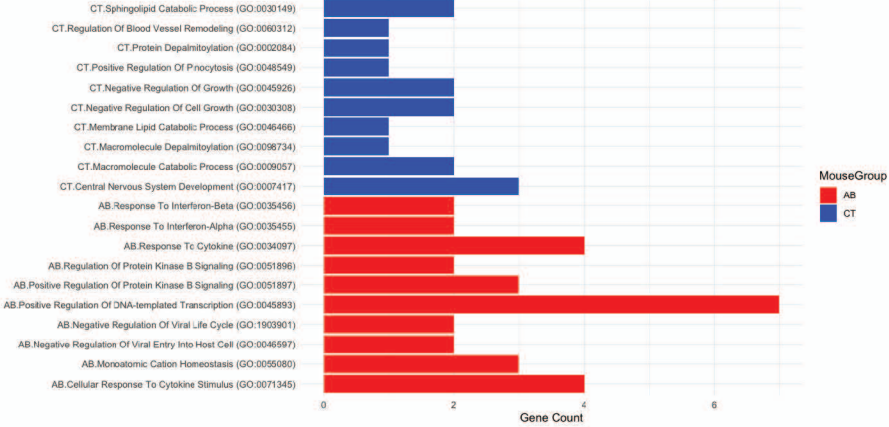

C

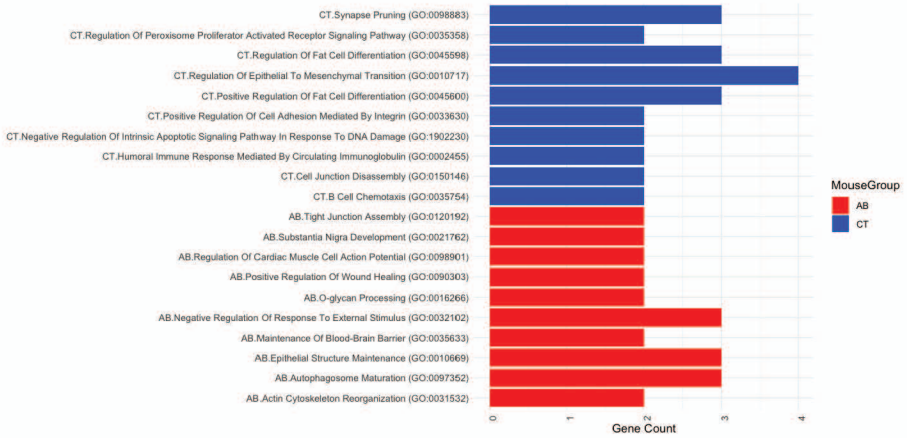

D

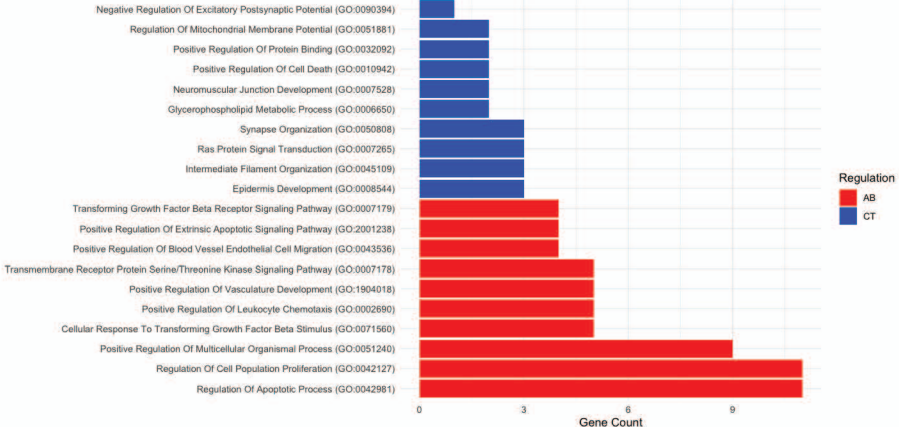

E

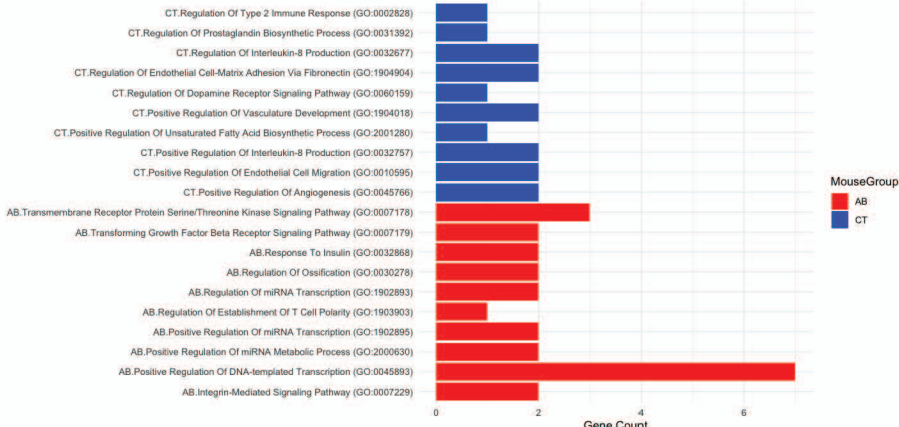

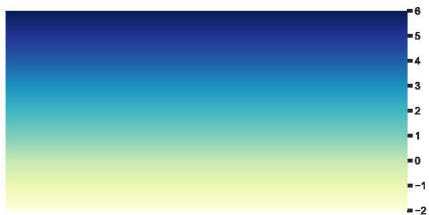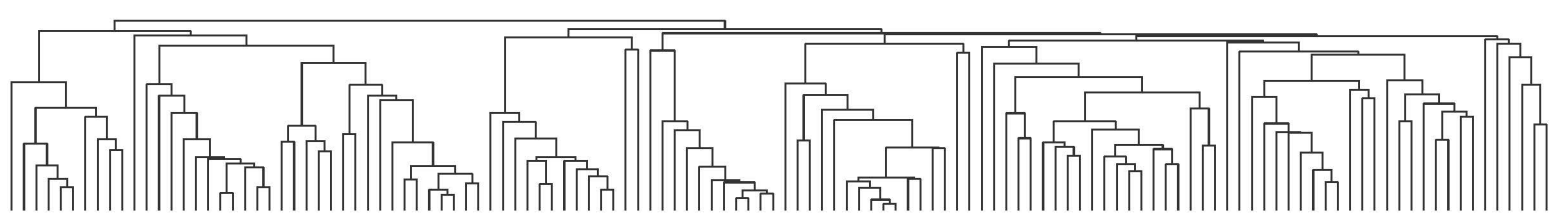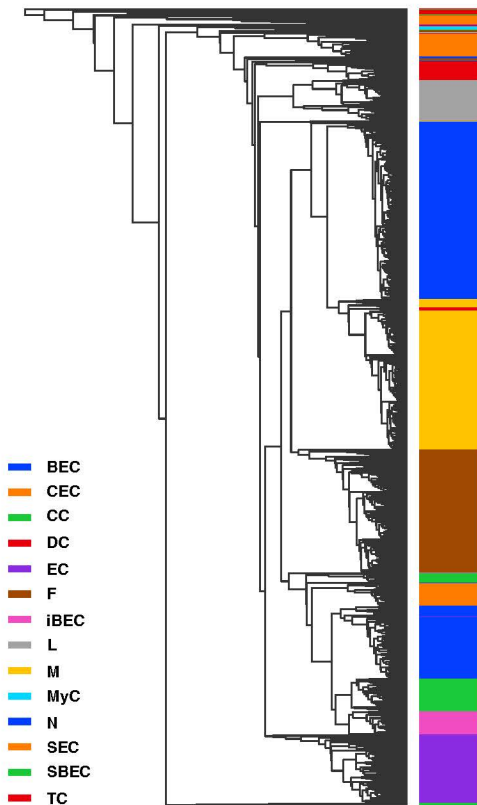

- BEC
- CEC
- CC
- DC
- EC
- F
- iBEC
- L
- M
- MyC
- N
- SEC
- SBEC
- TC

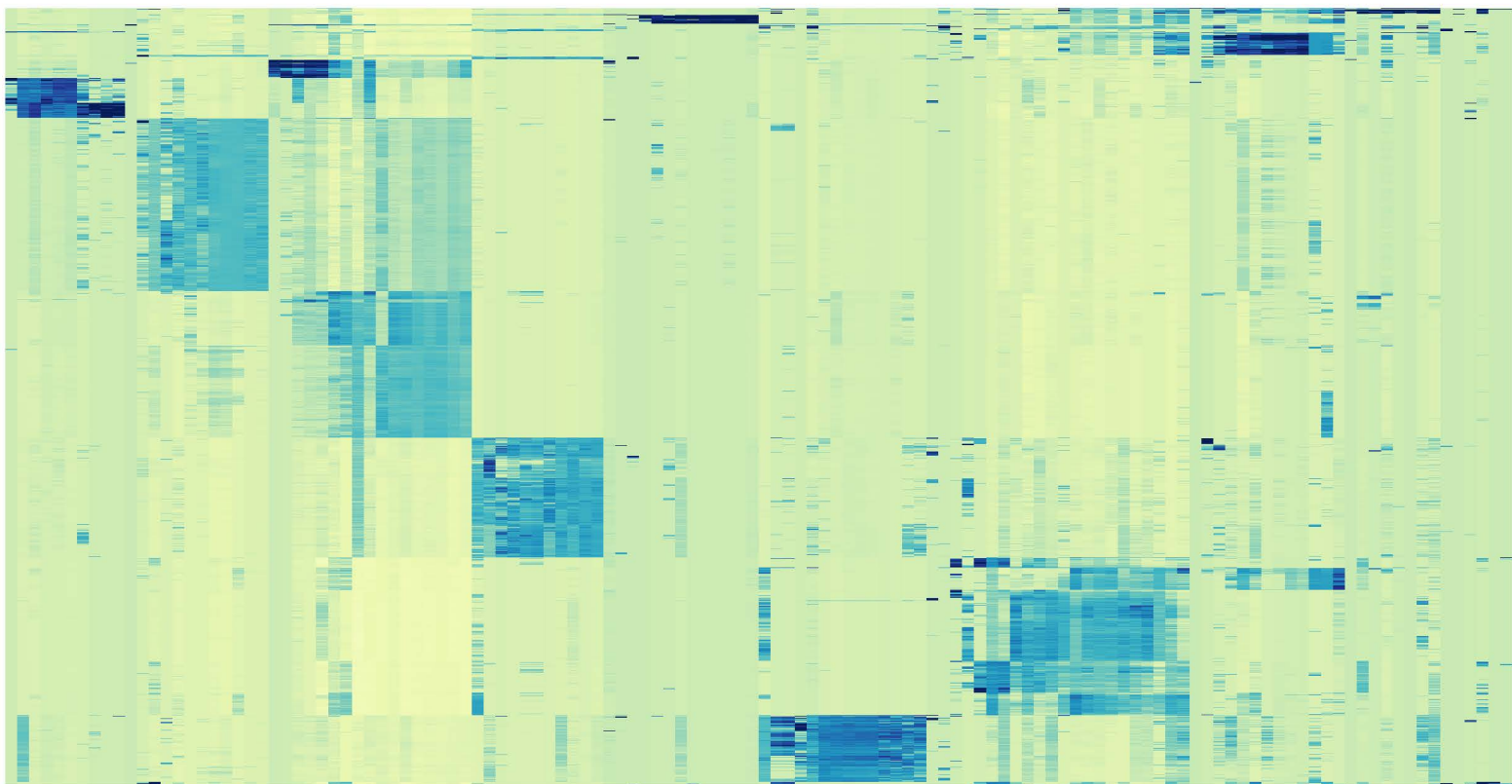



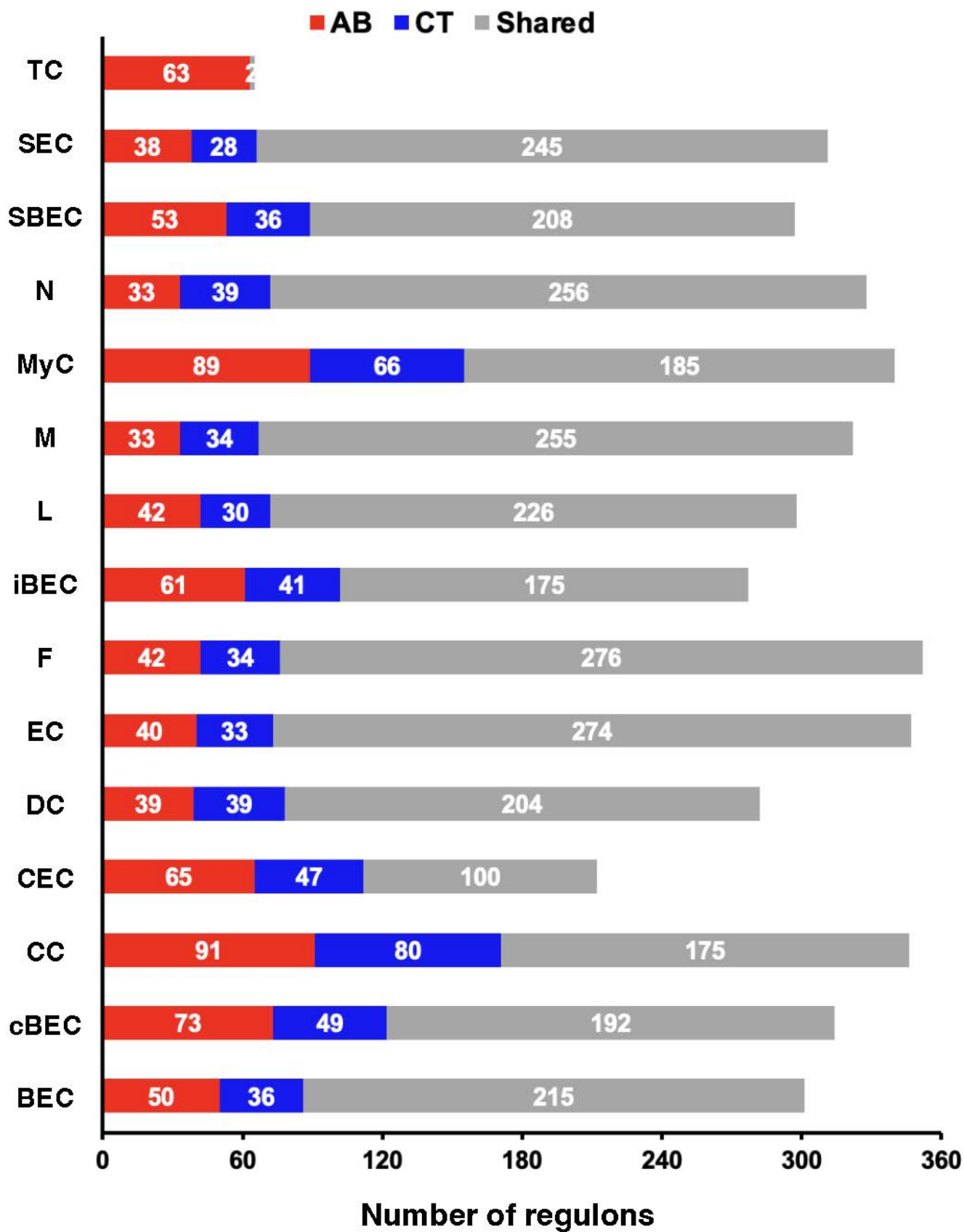

**A**

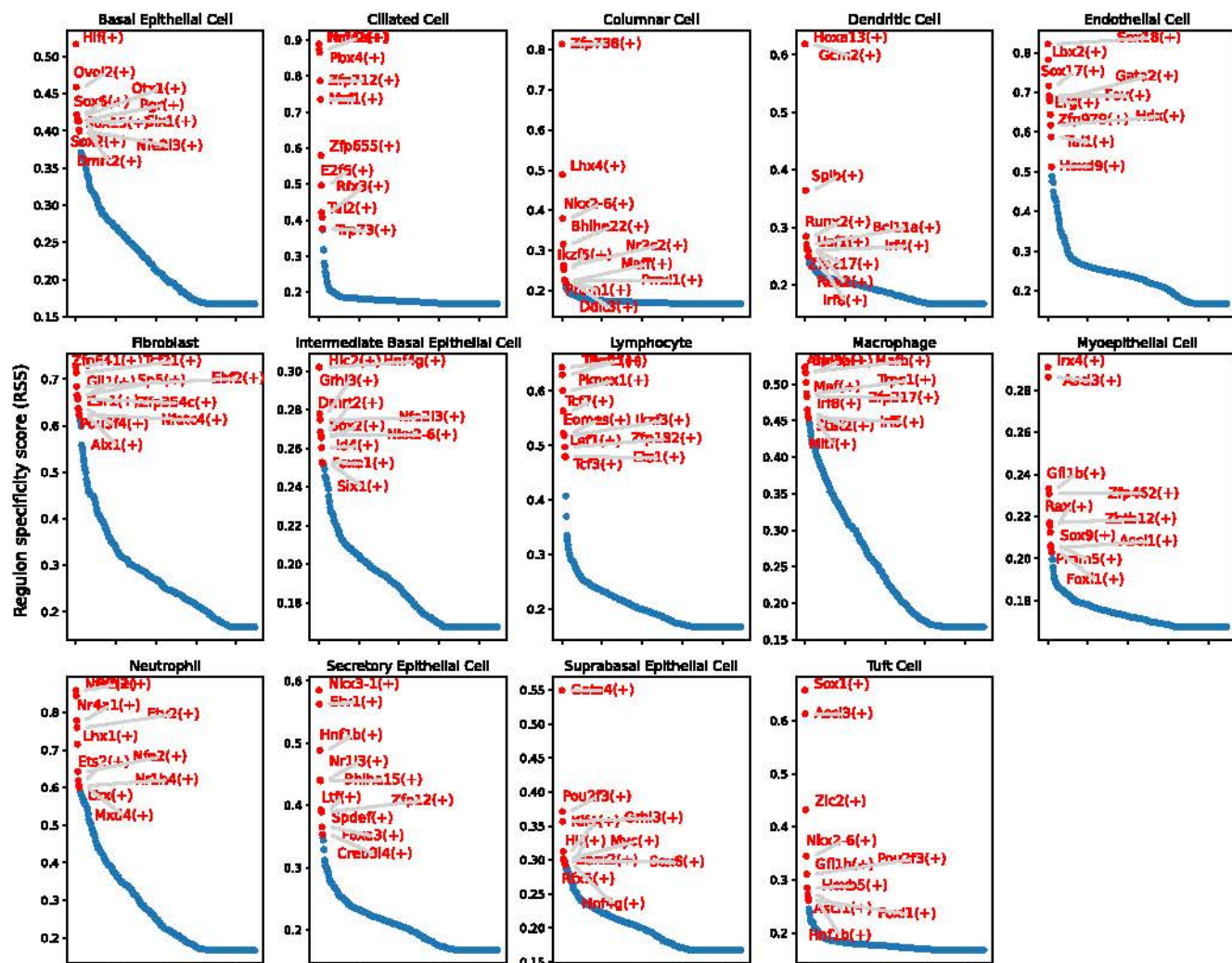

**B**

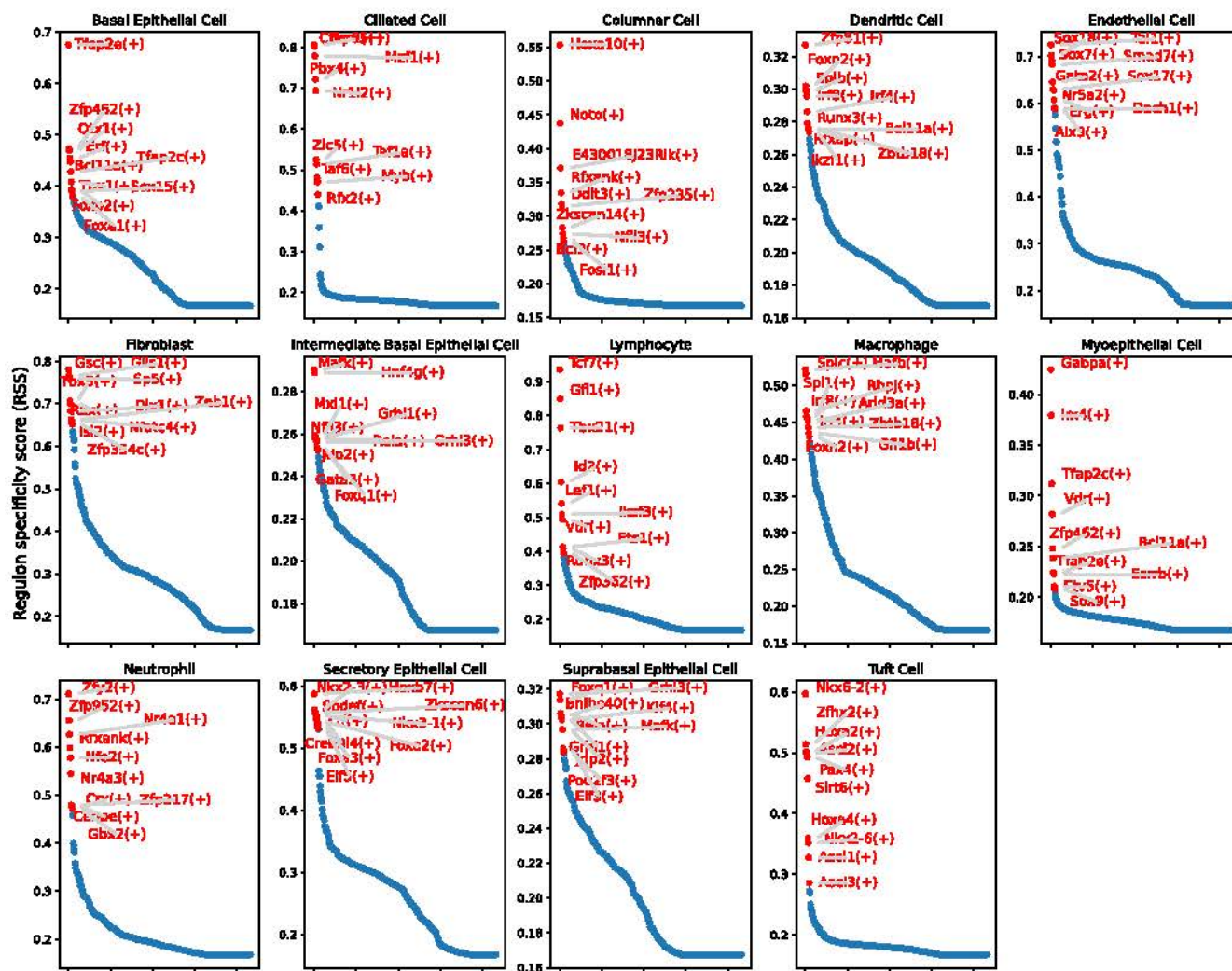

**A**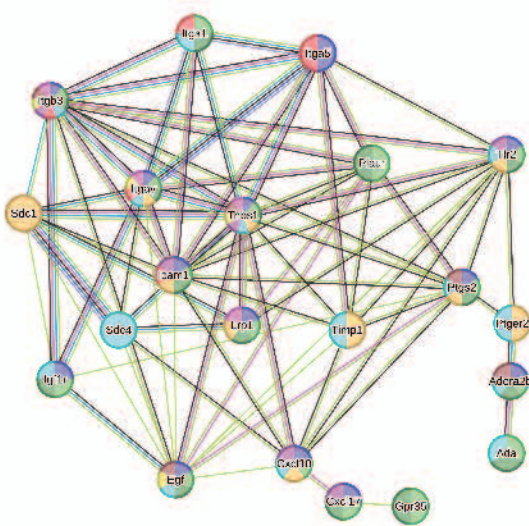**B**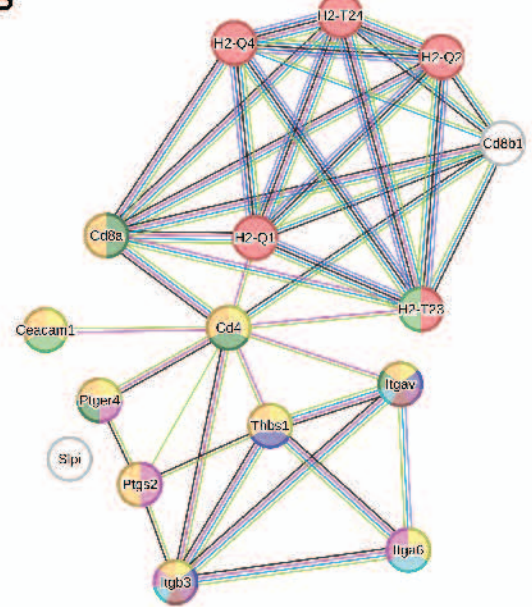**C**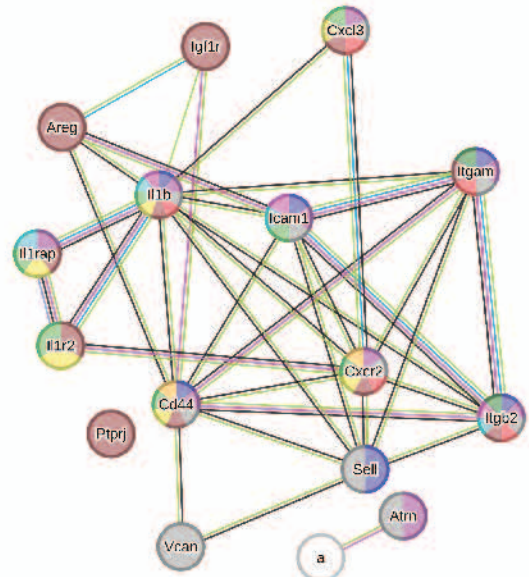**D**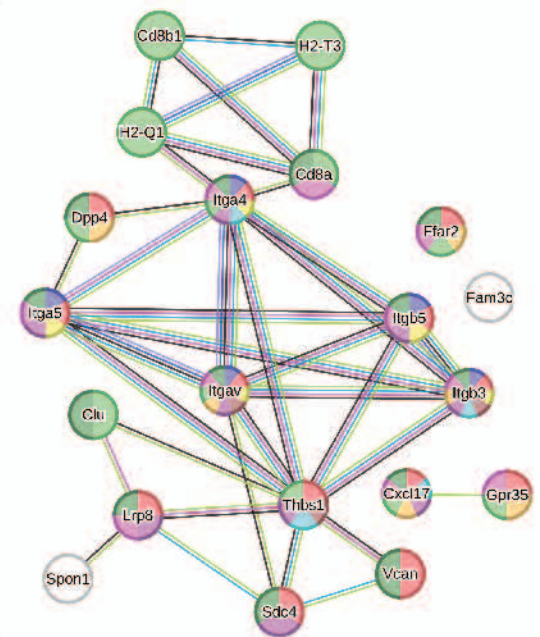**E**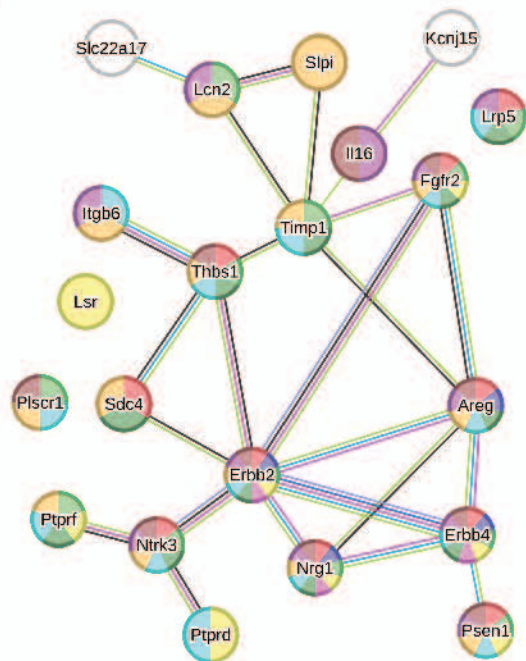**F**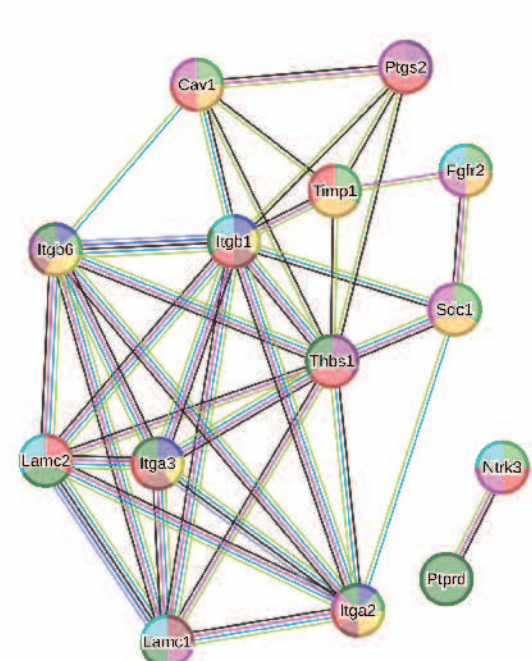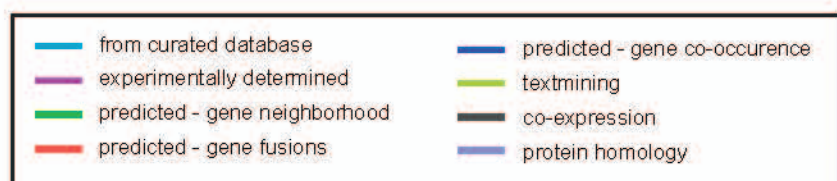

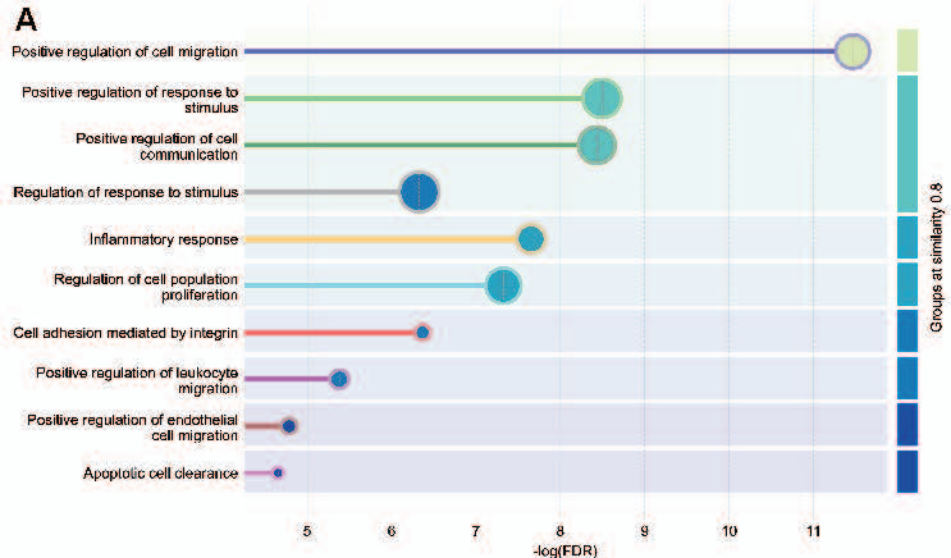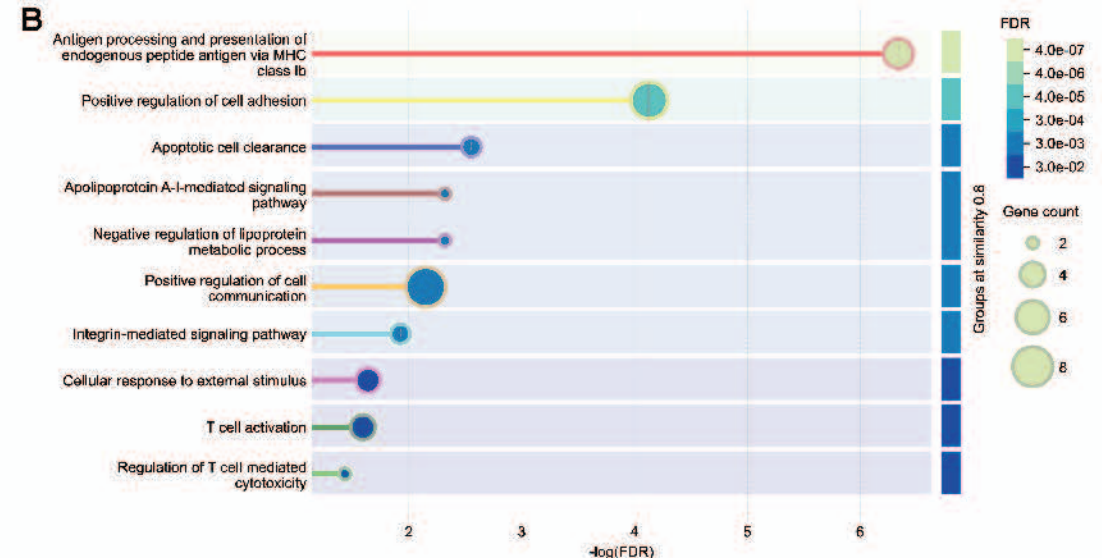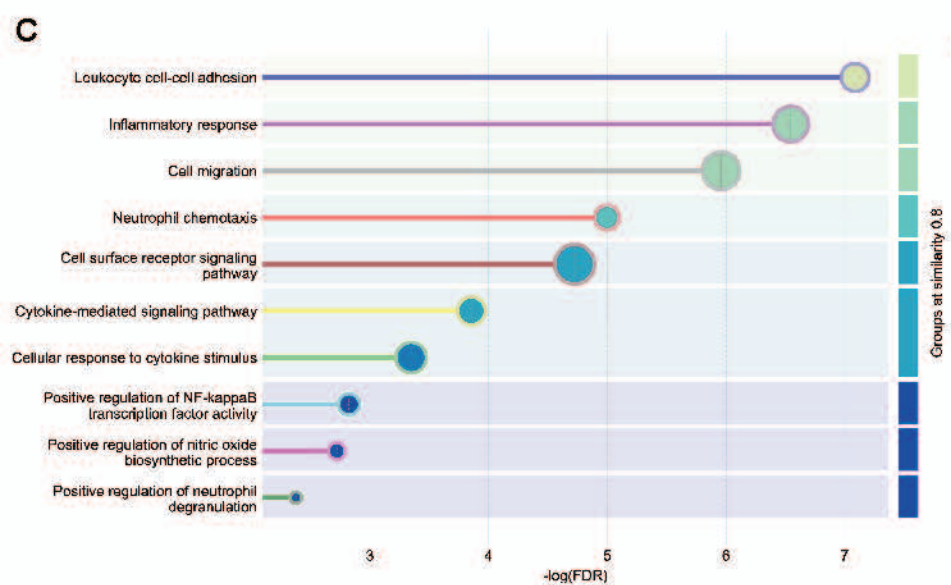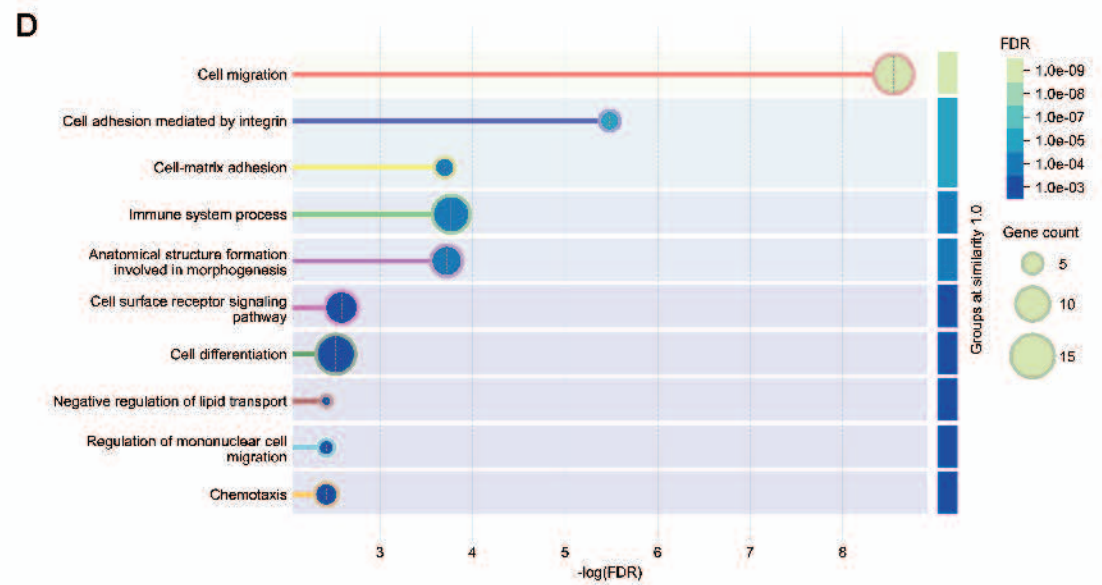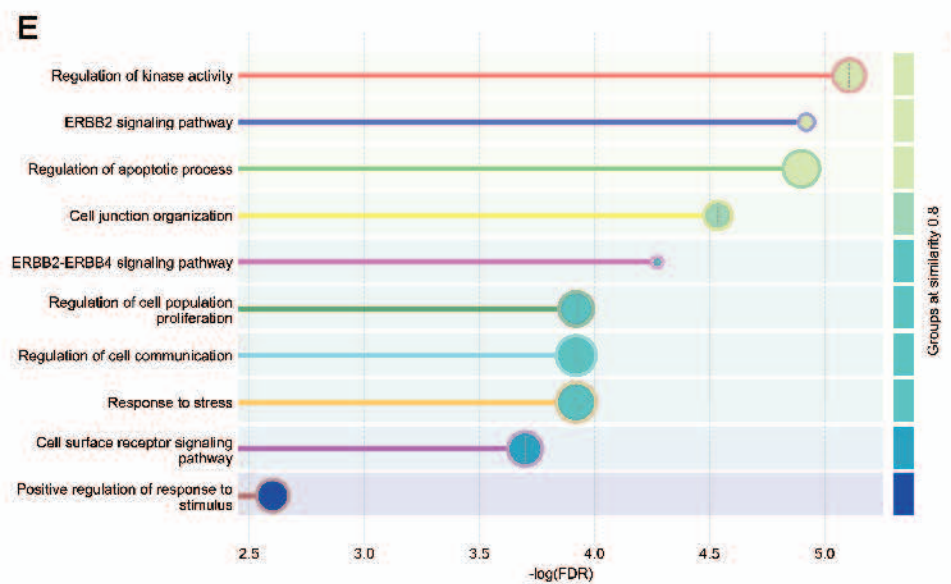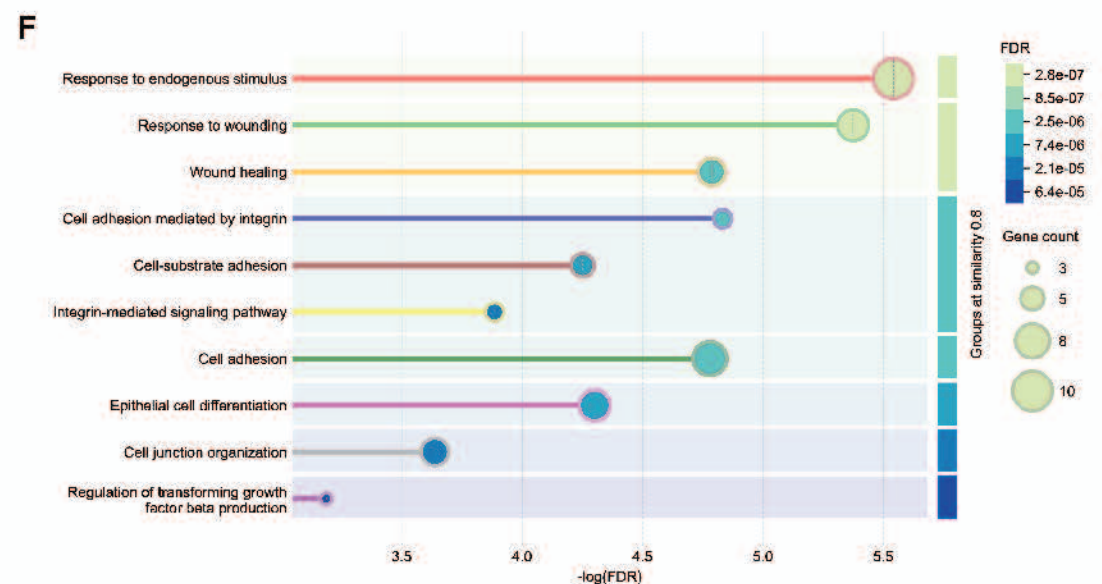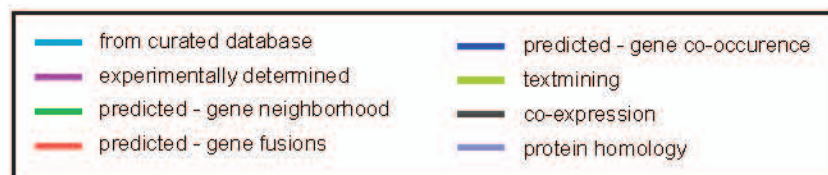

Supplement: Supplemental figures — Figures S1 to S10. [file msystems.01044-25-s0001.pdf]
