# Supplementary material for: Gut-larynx axis and its contribution to laryngeal immunity
Source: mSystems. 2025 Oct 7;10(11):e01044-25. doi: 10.1128/msystems.01044-25 (PMC12625719; doi:10.1128/msystems.01044-25)
Supplement: Legends — Supplemental figure legends. [file msystems.01044-25-s0002.docx]

**Figure S1. Phenotypic changes in mice following antibiotic treatment.**

1. Representation of the visible characteristics of antibiotic treated (AB) compared with control (CT) mice. M, F represents male, female.
2. Body weight of AB and CT mice after antibiotics treatment.
3. Representation of the cecum size, bacterial colony forming unit (CFU) in cecum and larynx in AB and CT mice. Laryngeal CFU exhibited no significant difference between conditions; cecum CFU was significantly decreased in AB mice according to student t-test (p = 0.05).
4. Principle coordinate (PCoA) analysis of β-diversity for gut and laryngeal microbiota in AB and CT mice using weighted UniFrac distance (1, 2) and Bray-Curtis distance (3, 4). PERMONOVA was applied to determine statistical difference in microbial community structure between groups (P < 0.001). Dotted contours indicate groups obtained by comparisons with PERMONOVA.

**Figure S2. Bias corrected analysis of composition (ANCOM-BC) of laryngeal or cecal microbiome between antibiotic treated (AB) and control (CT) mice.** Differential abundance of microbial taxa was assessed at genus level in larynx/cecum samples separately in QIIME 2 with default settings. The analysis accounts for compositionality and sampling bias in microbiome data, providing bias-corrected estimates of differential abundance. A significance threshold was set at an adjusted p-value (FDR-corrected) < 0.01. Taxa meeting this threshold were considered significantly different between groups and are denoted with an asterisk (*) within the bar.

**Figure S3. Laryngeal cell types and subtypes in antibiotic treated (AB) and control (CT) mice.**

1. UMAP plot of 23 laryngeal cell types found in antibiotics treated (AB) and control (CT) mice. BEC, iBEC, cBEC, C, CC, CEC, DC, EC, F, L, LEC, M, MC, MyC, N, P, RBC, SBEC, SC, SEC, SkMC, SMC, TC, and TEC represents basal epithelial cell, intermediate epithelial cell, cycling basal epithelial cell, chondrocyte, columnar cell, ciliated epithelial cell, dendritic cell, endothelial cell, fibroblast, lymphocyte, lymphatic endothelial cell, macrophage, muscle cell, myoepithelial cell, neutrophil, pericyte, red blood cell, suprabasal epithelial cell, Schwann cells, secretory epithelial cell, skeletal muscle cell, smooth muscle cell, tuft cell, and thymic epithelial cell, respectively.
2. Proportions of fibroblast (l, 4), macrophage (2, 5), and suprabasal epithelial cell (3, 6) subtypes in AB and CT mice using scCODA. UMAPs showing clustering of the subtypes of fibroblast, macrophages, and suprabasal epithelial cell (1, 2, 3); boxplots showing subtype proportion of these cell types in AB and CT mice. BEC, iBEC, cBEC, CC, CEC, DC, EC, F, L, M, MyC, N, SBEC, SEC, TC in each panel represents basal epithelial cell, intermediate epithelial cell, cycling basal epithelial cell, columnar cell, ciliated epithelial cell, dendritic cell, endothelial cell, fibroblast, lymphocyte, macrophage, myoepithelial cell, neutrophil, suprabasal epithelial cell, secretory epithelial cell, tuft cell, respectively.

**Figure S4. Horizontal barplots showing functional enrichment of differentially expressed genes (DEGs) in macrophage (A), dendritic cell (B), secretory epithelial cell (C), suprabasal epithelial cell (D), and endothelial cell (E) of antibiotic treated (AB) and control (CT) mice.** Average log2 fold change > 0.50, adjusted p-value < 0.05.

**Figure S5. Top 10 regulons identified in each major cell type in antibiotic treated (AB) mice.** BEC, cBEC, CEC, CC, DC, EC, F, iBEC, L, M, MyC, N, SEC, SBEC, and TC represents basal epithelial cell, cycling basal epithelial cell, ciliated epithelial cell, columnar cell, endothelial cell, fibroblast, intermediate basal epithelial cell, lymphocyte, macrophage, myoepithelial cell, neutrophile, secretory epithelial cell, subprabasal epithelial cell, and tuft cell. Generated by gathering the top 10 regulons for each cell type and plotting them in a heatmap. Top 10 regulons are selected based on their AUC score. Colors represent normalized AUC score.

**Figure S6. Top 10 regulons identified in each major cell types in control (CT) mice.** BEC, cBEC, CEC, CC, DC, EC, F, iBEC, L, M, MyC, N, SEC, SBEC, and TC represents basal epithelial cell, cycling basal epithelial cell, ciliated epithelial cell, columnar cell, endothelial cell, fibroblast, intermediate basal epithelial cell, lymphocyte, macrophage, myoepithelial cell, neutrophile, secretory epithelial cell, subprabasal epithelial cell, and tuft cell. Generated by gathering top 10 regulons for each cell type and plotting them in a heatmap. Top 10 regulons are selected based on their AUC score. Colors represent the normalized AUC score.

**Figure S7. Total number of regulons identified in each major cell type in antibiotic treated (AB) and control (CT) mice.** Red bars represent regulons unique to AB mice, blue bars indicate those unique to CT mice, and grey bars show regulons shared by both groups.

**Figure S8. Regulon specificity scores (RSS) for top 10 regulons in each major cell type in antibiotic treated (A) and control (B) mice.** RSS identifies regulons that are specific to particular clusters. RSS is calculated separately for each cell type using the Jensen–Shannon divergence; top 10 regulons are presented here.

**Figure S9. Visualization of ligand-receptor interactions (LRIs) within macrophages (A), lymphocytes (B), neutrophils (C), dendritic cells (D), secretory epithelial cells (E), and suprabasal epithelial cells (F).** Protein – protein interaction network displaying overview of the interactions between ligand and receptors identified in each primary emitter and receptor cell type. Analysis was performed at stringDB Nodes represent ligand/receptor genes; node color matches with GO term(s) line color(s) in the functional analysis dot plot, representing one or multiple biological processes associated with the genes. Edges represent protein-protein associations; edge color represent the method used to identify association.

**Figure S10. Functional analysis of ligand-receptor interactions (LRIs) within macrophages (A), lymphocytes (B), neutrophils (C), dendritic cells (D), secretory epithelial cells (E), and suprabasal epithelial cells (F).** Dot plot showing top 10 biological processes (GO terms) for protein network performed at stringDB. GO terms were grouped by similarity >= 0.8, false discovery = 0.001. Dot size represents count of genes associated with corresponding GO term, line color is unique to each GO term and matches with the node color in the protein network on the left.
