# Supplementary material for: Gut-larynx axis and its contribution to laryngeal immunity
Source: mSystems. 2025 Oct 7;10(11):e01044-25. doi: 10.1128/msystems.01044-25 (PMC12625719; doi:10.1128/msystems.01044-25)
Supplement: Supplemental table — Table S1. [file msystems.01044-25-s0003.docx]

**Table S1. Facts about antibiotics used.**

| **Antibiotic** | **Gut Absorption** | **Systemic Effect** | **Target**  **Bacteria** | **Target**  **Organ** | **References** |
| --- | --- | --- | --- | --- | --- |
| Ampicillin (A) | Moderate | Yes (good systemic effect) | Broad: Gram-positive (e.g., Streptococcus, Enterococcus) & some Gram-negative (e.g., Haemophilus influenzae) | Respiratory tract, urinary tract, gastrointestinal tract | Bush et al. 2016 Moellering et al. 1989 |
| Neomycin (N) | Poor (oral formulation) | Minimal (limited to gut) | Broad: Gram-negative (aerobic) & some Gram-positive bacteria, excluding anaerobes | Gut (pre-surgical prep), skin (topical) | Weinstein et al. 1963. Murray et al. 1998 |
| Vancomycin (V) | Poor (oral formulation) | Limited (oral: gut only; IV: systemic) | Narrow: Gram-positive (aerobic & anaerobic) like MRSA & Clostridium difficile | Gut (oral), systemic (IV: skin, heart, lungs) | Levine et al. 2006. Pogue et al. 2011 |
| Metronidazole (M) | High (oral formulation) | Yes (good systemic effect) | Narrow: Anaerobic bacteria (Clostridium, Bacteroides, Helicobacter pylori) & some protozoa | Gut, genitourinary tract, and liver | Brook et al. 1994. Freeman et al. 1997. |
